# Comparative effects of exercise modalities on depression, anxiety, and stress in university students: a systematic review and network meta-analysis of randomized controlled trials

**DOI:** 10.3389/fpubh.2026.1795525

**Published:** 2026-05-29

**Authors:** Zheng Zhao, Ting Li, Huamin Lai, Renjun Yang

**Affiliations:** 1 Physical Education Institute, Anyang Normal University, Anyang, Henan, China; 2 Officers College of PAP, Chengdu, China; 3 Department of Rehabilitation, West China Hospital Sichuan University Jintang Hospital. Jintang First People's Hospital, Chengdu, China

**Keywords:** anxiety, depression, exercise, network meta-analysis, randomized controlled trials, stress, university students

## Abstract

**Objective:**

To compare the effects of different exercise interventions on depression, anxiety, and stress in university students and to identify outcome-specific patterns and relevant prescription features using network meta-analysis.

**Methods:**

A systematic review and network meta-analysis of randomized controlled trials was conducted following PRISMA guidelines. PubMed, Embase, Cochrane Central Register of Controlled Trials, Web of Science, and Google Scholar were searched from inception to 31 October 2025. Eligible studies included university students aged 18 years or older, structured exercise interventions lasting at least 4 weeks, and validated measures of depressive symptoms, anxiety symptoms, or perceived stress. Pairwise random-effects meta-analyses and random-effects network meta-analyses were performed. Effects were summarized as standardized mean differences (SMDs) with 95% confidence intervals (95% CIs), and treatments were ranked using the surface under the cumulative ranking curve.

**Results:**

Twenty-five RCTs involving 2,127 students were included. Compared with control conditions, exercise was associated with improvements in depression (SMD −1.07, 95% CI −1.38 to −0.75; *I*^2^ = 87.6%), anxiety (SMD −0.69, 95% CI −1.00 to −0.38; *I*^2^ = 87.1%), and stress (SMD −0.45, 95% CI −0.73 to −0.16; *I*^2^ = 75.2%). These effects should be interpreted cautiously due to substantial heterogeneity and variation in outcome measures. Network meta-analysis indicated outcome-specific patterns: mind–body exercise and aerobic exercise were most consistently associated with favorable outcomes for depression; mind–body exercise showed clearer associations for anxiety, whereas team sports and resistance-based approaches appeared favorable but were supported by limited evidence; aerobic exercise was linked to the strongest comparative effect for stress. Exploratory subgroup analyses suggested that sessions of 30–59 min were more frequently associated with benefits for depression and anxiety, whereas stress benefits were more often observed with sessions of 30 min or longer. Moderate to vigorous intensity and higher weekly frequency were associated with more favorable anxiety and stress outcomes, although these findings should be considered exploratory rather than definitive.

**Conclusion:**

Exercise interventions are associated with improvements in depression, anxiety, and stress in university students, with outcome-specific patterns varying by modality. Exploratory findings suggest that time-efficient, moderate to vigorous exercise could inform future research on student mental health promotion.

**Systematic review registration:**

https://www.crd.york.ac.uk/PROSPERO/view/CRD420261379734.

## 1 Introduction

Mental health in university students is commonly conceptualized as psychological wellbeing and functional adaptation, with depressive symptoms, anxiety symptoms, and perceived stress representing key and often co-occurring domains (1). Global syntheses suggest that these problems are widespread: one large systematic review and meta-analysis reported pooled prevalences of 33.6% for depressive symptoms and 39.0% for anxiety symptoms among college students (2). Stress is also highly prevalent in university populations; for example, a recent meta-analysis reported an overall prevalence of perceived stress of 64.72% among university students in Africa, highlighting the potential magnitude of burden in resource constrained settings (3). These conditions are associated with impaired academic functioning, reduced social participation, and increased likelihood of persistence into adulthood, thereby shaping long-term health and socioeconomic trajectories. At the societal level, depression and anxiety are linked to substantial productivity losses, with estimates of around US$1 trillion per year (4). Collectively, these data support the urgency of identifying scalable and acceptable interventions to improve mental health in university students.

Current approaches to student mental health management typically include pharmacotherapy, psychological therapies such as cognitive behavioral therapy (CBT), counselling services, and digital or campus-based support programmes (5, 6). Although these strategies can be effective, real-world implementation is often constrained by limited-service capacity, delayed access, stigma, and adherence challenges (7). Medication may also be limited by adverse effects and low acceptability in younger adults, while psychotherapy requires sustained engagement and trained providers that may be difficult to deliver at scale in many university systems (8). Exercise and broader physical activity (PA) interventions have therefore received increasing attention as pragmatic options that can be integrated into student routines. In addition to potential psychological benefits, PA may improve sleep and physical health, facilitate social connection, and encourage health behaviors that can extend beyond the intervention period, aligning with the preventive and health promotion priorities of university settings (9).

A growing body of randomized controlled trials (RCTs) suggests that exercise-based interventions can improve depressive and anxiety symptoms in university students, with benefits also reported for stress outcomes. Meta-analysis evidence focused on undergraduate populations indicates that PA interventions can produce measurable improvements in mental health, although effect sizes vary and findings are influenced by differences in intervention design and comparator conditions (9, 10). Evidence from broader clinical populations further supports exercise as an effective treatment component for depression, strengthening biological plausibility and clinical relevance (11). However, important gaps remain for translation into university practice. Many previous syntheses have pooled heterogeneous exercise programmes into a single exposure, examined a single outcome, or prioritized intensity or dose without offering clear comparative evidence across the full set of outcomes most relevant to students. Even when comparative approaches are used, inconsistent intervention categorization, limited head-to-head trials, and variability in baseline symptom status and key prescription features complicate interpretation and limit actionable recommendations.

Therefore, the present study aimed to synthesize RCT evidence on exercise interventions for university students and to compare their relative effects on depression, anxiety, and stress. By clarifying comparative benefits across outcomes within a single evidence framework, the findings are intended to support feasible and evidence informed mental health promotion in university settings and to guide priorities for future trials.

## 2 Methods

### 2.1 Protocol and reporting

This systematic review and network meta-analysis was conducted in accordance with the Preferred Reporting Items for Systematic Reviews and Meta Analyses (PRISMA) 2020 statement and its extension for network meta-analyses (12). The review was retrospectively registered in the International Prospective Register of Systematic Reviews, PROSPERO, under registration number CRD420261379734.

### 2.2 Search strategy

PubMed, Embase, Cochrane Central Register of Controlled Trials (CENTRAL), Web of Science, and Google Scholar were searched from inception to 31 October 2025 without language restrictions. Search terms combined controlled vocabulary and free text related to (a) university or college students (for example, “college student,” “university student,” “undergraduate,” “graduate student”), (b) exercise or physical activity (for example, “exercise,” “physical activity,” “aerobic training,” “resistance training,” “high intensity interval training,” “yoga,” “team sport”), and (c) psychological outcomes (for example, “depression,” “anxiety,” “stress”). The full search strategy for each database, including Boolean operators and controlled vocabulary, is detailed in Supplementary material, Section 2 (Search Strategy). Reference lists of eligible studies and relevant reviews were hand searched. Two reviewers independently screened titles and abstracts, then full texts. Disagreements were resolved by discussion or third reviewer adjudication.

### 2.3 Eligibility criteria and study selection

Studies were included if they met all prespecified criteria: (a) participants were university students (undergraduate or postgraduate) aged 18 years or older; mixed samples were eligible only when student data were separable or constituted at least 80% of the sample; (b) interventions were structured exercise programmes lasting at least 4 weeks; modalities were classified as aerobic exercise (AE), resistance training (RT), concurrent training combining aerobic and resistance components (CT), high intensity interval training (HIIT), mind–body exercise (MBE; for example, yoga, tai chi, qigong), or team sports (TS), based on the dominant component; (c) comparators were restricted to eligible non-exercise control conditions, including usual care, usual lifestyle, no intervention, wait-list, or non-therapeutic attention or health education controls. For consistency, these groups were collectively treated as control conditions because they did not involve structured exercise exposure. Comparator conditions reflecting routine university physical education were considered usual curriculum comparators when they were not prescribed or delivered as part of the trial intervention. Because such comparators may still include some physical activity exposure, their influence was examined in sensitivity analysis. Comparators involving structured active non-exercise interventions, such as mindfulness meditation, psychotherapy, relaxation training, or other behavioral therapeutic programs, were not classified as control conditions. Such studies were included only if they contained a separate eligible non-exercise control arm; otherwise, they were excluded from the exercise-versus-control quantitative synthesis; (d) outcomes included at least one validated measure of depressive symptoms, anxiety symptoms, or perceived stress assessed at intervention end. Depression scales included Beck Depression Inventory (BDI or BDI II), Center for Epidemiologic Studies Depression Scale (CES D or CESD R), Depression Anxiety Stress Scale 21 depression subscale, Depression Status Inventory, General Health Questionnaire 28, Hospital Anxiety and Depression Scale depression subscale, Patient Health Questionnaire 4 or 9, Symptom Checklist 90 depression dimension, or Self Rating Depression Scale. Anxiety scales included Beck Anxiety Inventory, Depression Anxiety Stress Scale 21 anxiety subscale, Generalized Anxiety Disorder 7 item scale, General Health Questionnaire 28, Hospital Anxiety and Depression Scale anxiety subscale, Hamilton Anxiety Scale, Patient Health Questionnaire 4, State Anxiety Inventory, Self Rating Anxiety Scale, Symptom Checklist 90 anxiety dimension, State Trait Anxiety Inventory, or its 6-item version. Stress scales included Chinese Perceived Stress Scale, Depression Anxiety Stress Scale 21 stress subscale, Perceived Stress Scale, Perceived Stress Scale 10 item version, or Student Life Stress Inventory; (e) design was a published randomized controlled trial (parallel or cluster). Cross over trials contributed only first period data. English language peer reviewed full texts were required.

Studies were excluded if (a) only acute single session exercise was tested, (b) exercise was combined with non-exercise co interventions such as diet, medication, or psychotherapy without separable effects, (c) data were insufficient to compute effect sizes and could not be obtained from authors, or (d) full text was unavailable, or the report was a protocol, abstract, dissertation, or non-human study. Multiple reports from the same trial were collated and the most complete dataset was used.

### 2.4 Data extraction and intervention coding

Records were managed in EndNote X9. Two reviewers independently extracted (a) publication and trial details, (b) participant characteristics, (c) intervention and comparator prescriptions including session duration, weekly frequency, total duration, supervision, and adherence, and (d) outcome means and standard deviations at baseline and post intervention. Standard deviations were derived from standard errors, confidence intervals, t values, ranges, or *p* values using Cochrane guidance. Where required, study authors were contacted at least four times over 6 weeks. Exercise intensity was coded from reported physiological targets or estimated metabolic equivalents using the Compendium of Physical Activities, and grouped as light, moderate, or vigorous (13).

### 2.5 Risk of bias assessment

Risk of bias was assessed independently by two reviewers using the Cochrane revised risk of bias tool (RoB 2) across five domains: randomization process, deviations from intended interventions, missing outcome data, outcome measurement, and selective reporting (14). Detailed study-level RoB judgments with rationale are provided in Supplementary material, Section 4 (Supplementary Table S4). Disagreements were resolved by consulting a third reviewer.

### 2.6 Statistical analysis

#### 2.6.1 Pairwise meta-analysis

All analyses used Stata 17. Random effects models were applied. Effect sizes were calculated as standardized mean differences (SMDs) with 95% confidence intervals (95% CIs). Primary analyses used post intervention values; change scores were used when necessary. If change score standard deviations were missing, they were imputed as: SD_change = √(SD_baseline^2^ + SD_post^2^–2 × R × SD_baseline × SD_post), with R set to 0.5 and varied in sensitivity analyses. Negative SMDs indicated symptom reduction relative to comparator. Heterogeneity was evaluated using Cochran *Q* and *I*^2^ (25, 50, and 75% indicating low, moderate, and high heterogeneity). Because multiple validated instruments with different scoring ranges were pooled, SMDs were interpreted as standardized relative effect estimates rather than directly equivalent clinical changes on any single scale. Sensitivity analyses excluded outliers whose 95% CI did not overlap the pooled 95% CI and removed trials at high risk of bias. For outcomes involving a routine physical education comparator, the pairwise analyses were also repeated after excluding that study to assess the influence of possible usual curriculum physical activity exposure. Prespecified subgroup analyses examined baseline symptom status, intensity, session duration, frequency, and intervention length; where possible, meta regression explored these modifiers. These analyses were considered exploratory and were interpreted as hypothesis generating rather than causal. Publication bias was assessed by funnel plots and Egger tests when at least 10 studies contributed (15). When Egger testing suggested potential small-study effects, an exploratory trim-and-fill sensitivity analysis was additionally performed to examine the possible impact on the pooled pairwise estimate.

#### 2.6.2 Network meta-analysis

Random effects network meta-analyses were conducted separately for depression, anxiety, and stress. Nodes represented AE, RT, CT, HIIT, MBE, TS, and control; edges represented direct randomized comparisons. The network meta-analysis was conducted under a frequentist framework using the Stata 17 network suite. Consistency and inconsistency models were fitted in a contrast-based multivariate random-effects meta-analysis/meta-regression framework using mvmeta. Therefore, no Bayesian prior distributions were specified. Transitivity was assessed by comparing the distribution of key potential effect modifiers across treatment comparisons, including mean age, baseline symptom severity where available, exercise intensity in METs, session duration, weekly frequency, and intervention length. No single numerical threshold was prespecified; comparability was judged from the extent of overlap in these characteristics across comparisons. To further support this assessment, exploratory single-covariate network meta-regression analyses were performed for the main available covariates. Because several intervention nodes were informed by relatively few studies and some covariates were incompletely reported, these analyses were interpreted as exploratory rather than confirmatory. Consistency was tested globally with the design by treatment interaction model and locally with node splitting. A common between-study heterogeneity variance was summarized as *τ*^2^, and treatment ranking probabilities and SUCRA values were generated within the same frequentist framework. Certainty of evidence for the main network comparisons was additionally assessed using a structured GRADE-informed approach adapted for network estimates. Starting from high certainty for randomized evidence, we considered risk of bias, inconsistency, indirectness, imprecision, and publication bias. Treatment rankings were interpreted together with the certainty of the underlying comparison estimates rather than as stand-alone evidence. The resulting judgments are presented in Supplementary material, Section 10. Network heterogeneity was summarized using the common between-study variance (*τ*^2^), and approximate network *I*^2^ values were used to aid interpretation of heterogeneity across outcome-specific networks. Treatments were ranked using the surface under the cumulative ranking curve (SUCRA), with higher values indicating greater likelihood of being most effective. Two-sided *p* values below 0.05 were considered statistically significant.

## 3 Results

### 3.1 Study selection and characteristics

**Figure 1:**
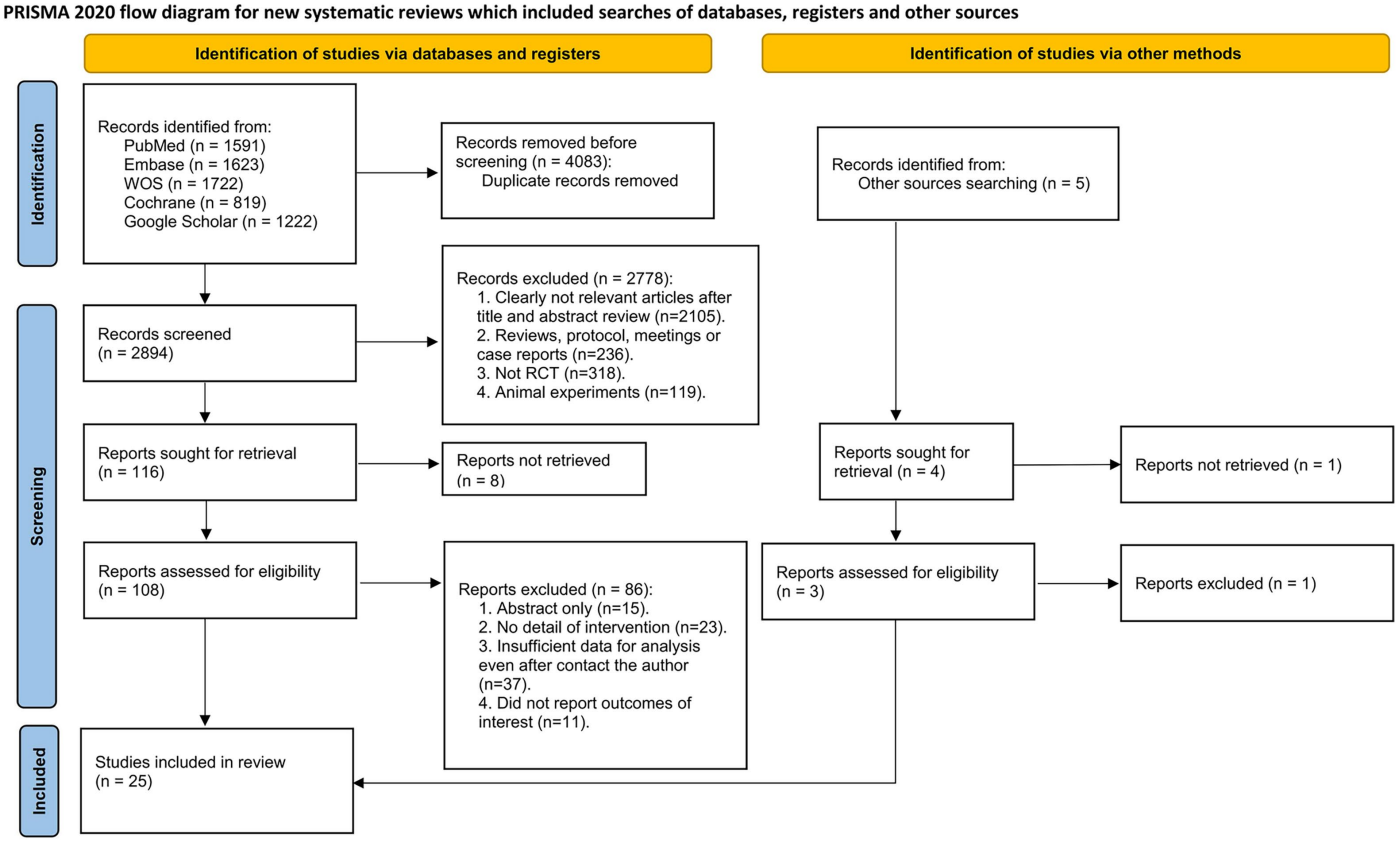
PRISMA flow diagram of study selection.

The search identified 6,977 records. After removal of 4,083 duplicates, 2,894 titles and abstracts were screened. A total of 2,778 were excluded, and 116 full texts were assessed. Twenty-five RCTs involving 2,127 university students were included (Figure 1) (16–40), with detailed characteristics provided in Supplementary material, Section 3 (Supplementary Tables S3.1–S3.25: Study design, participants, intervention prescription, completion, adverse events, and outcomes).

The trials were published between 1987 and 2024, with a median publication year of 2021. Ten studies were conducted in China and five in the United States. Ten trials used single-blind designs and fifteen were open-label. Sample sizes ranged from 18 to 679 participants (median 53). Mean participant age ranged from 18.4 to 26.1 years (median 20.8). Twelve trials reported baseline BMI (20.7–27.4 kg/m^2^; median 22.0). Baseline depression scores were reported in 22 studies, and 16 of these included students with depression at baseline.

Four studies were three-arm trials; the remaining 21 were two-arm designs. Regarding exercise modality, 12 studies evaluated MBE, 8 evaluated AE, 2 evaluated CT, 3 evaluated HIIT, 2 evaluated RT and 2 evaluated TS. Exercise intensity ranged from 3.0 to 9.6 METs (median 5.3). Single-session duration ranged from 10 to 90 min (median 40). Weekly frequency ranged from 1 to 5 sessions (median 3), and intervention duration ranged from 2 to 16 weeks (median 8). Adherence and completion reporting were inconsistent, and limited information on adverse events was provided.

### 3.2 Meta-analysis

Single-covariate NMA results are presented in Supplementary material, Section 7. For depression, older mean age was associated with weaker effects, whereas longer sessions showed stronger effects. For anxiety, longer session duration and intervention length were beneficial. For stress, older age was associated with weaker effects; exercise intensity and session duration showed borderline associations. These findings should be interpreted cautiously due to limited data for some nodes.

Certainty of evidence for the main network comparisons was generally low to very low (Supplementary material, Section 10). The comparisons of mind–body exercise versus control and aerobic exercise versus control for depression, as well as aerobic exercise versus control for stress, were rated as low certainty, mainly because of heterogeneity and imprecision. The certainty of evidence for the main anxiety comparisons was judged as very low because of substantial heterogeneity, sparse evidence, imprecision, and publication bias. These judgments indicate that treatment rankings should be interpreted cautiously. Accordingly, modality rankings were interpreted as tentative comparative signals rather than definitive evidence for selecting a specific exercise modality.

#### 3.2.1 Depression

**Figure 2:**
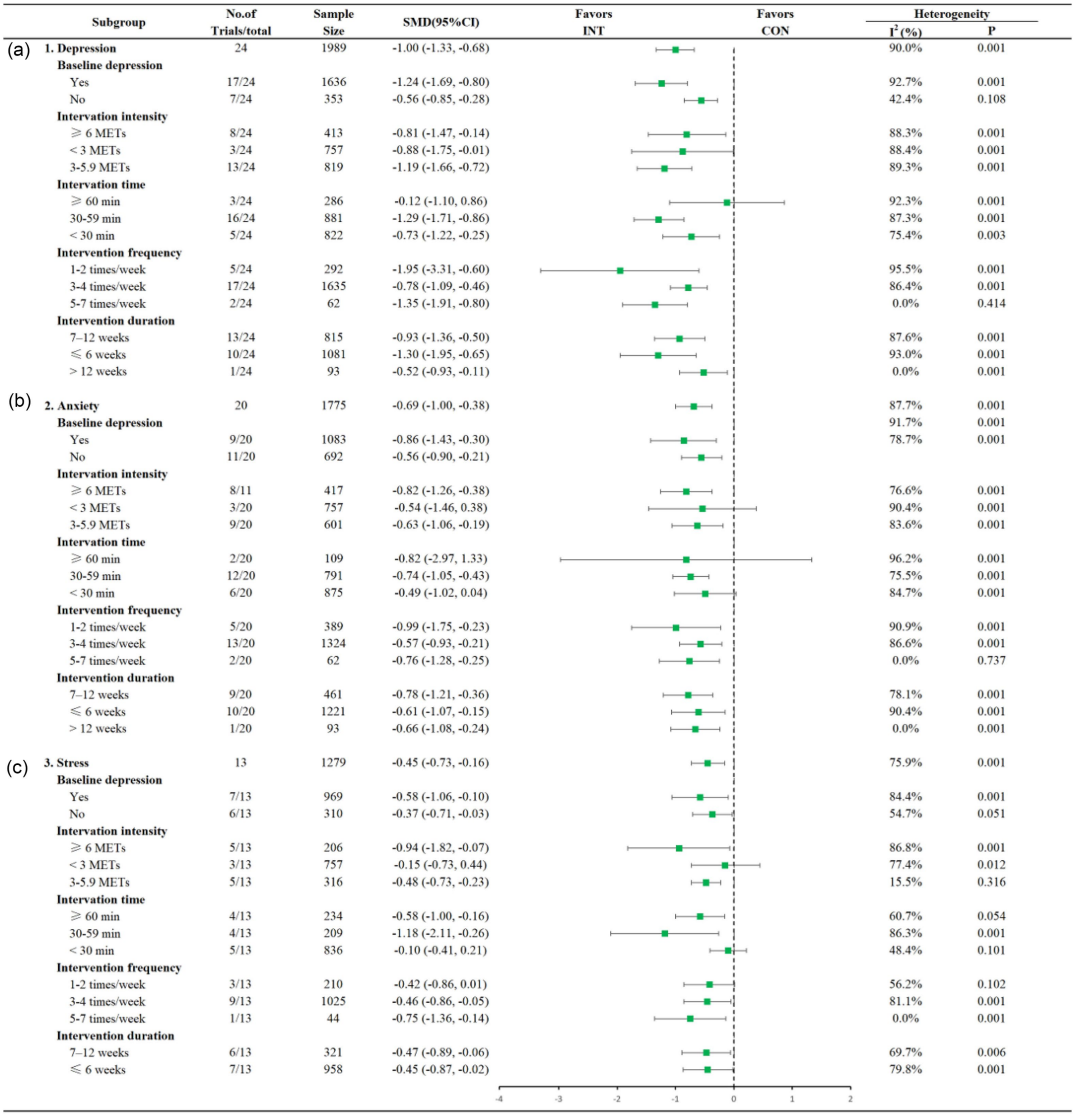
Pairwise meta-analyses of exercise interventions on **(a)** depression, **(b)** anxiety, and **(c)** stress in university students.

Twenty-one studies (*n* = 1,754) reported depression outcomes. Exercise significantly reduced depressive symptoms compared with control (SMD = −1.07; 95% CI −1.38 to −0.75; *I*^2^ = 87.6%; Figure 2). Sensitivity analyses are reported in Supplementary material, Section 6 (Figure 6.1) and showed stable results. In an additional analysis excluding Zhang et al. (36), which used routine physical education as the comparator, the pooled estimate remained similar (SMD = −1.08; 95% CI −1.42 to −0.75; *I*^2^ = 89.0%; Supplementary Table S6.1), with no change in direction or statistical significance.

Exploratory subgroup analyses showed significant differences by intervention time. Sessions of 30–59 min had the largest effect (SMD = −1.29; 95% CI −1.71 to −0.86). Sessions shorter than 30 min also improved depression (SMD = −0.73; 95% CI −1.22 to −0.25). Sessions of at least 60 min were not significant (SMD = −0.45; 95% CI −1.75 to 0.85). All subgroups defined by baseline depression, intensity, weekly frequency and duration showed significant reductions.

**Figure 3:**
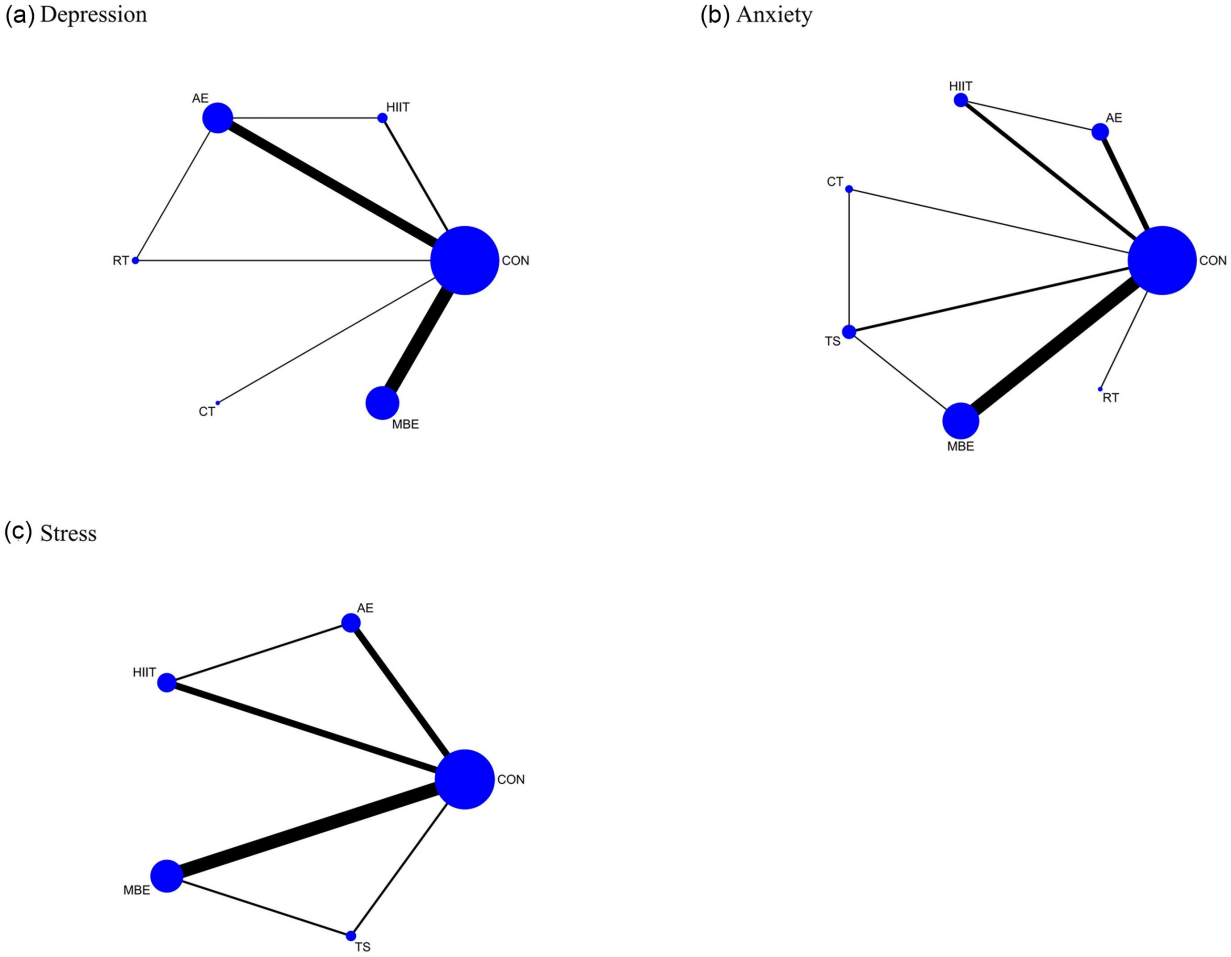
Network geometry of randomized comparisons among exercise modalities for **(a)** depression, **(b)** anxiety, and **(c)** stress in university students. Node size reflects the number of participants, and edge thickness represents the number of direct comparisons.

**Figure 4:**
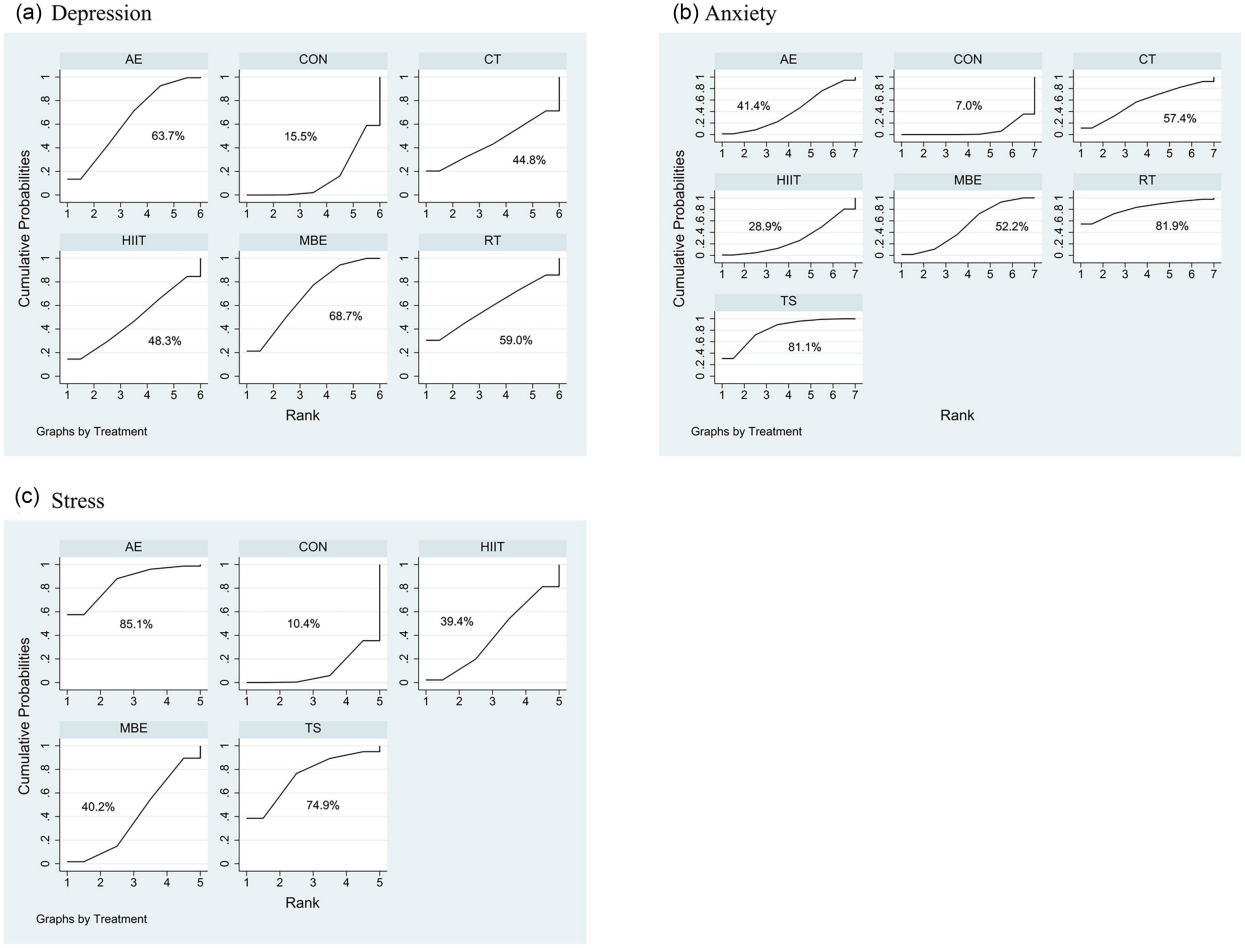
Surface under the cumulative ranking curve (SUCRA) plots ranking exercise modalities for **(a)** depression, **(b)** anxiety, and **(c)** stress in university students.

The NMA structure is shown in Figure 3a. Network *I*^2^ of 88.1% and *τ*^2^ of 0.53. SUCRA rankings (Figure 4a) indicated that MBE (68.7%), AE (63.7%) and RT (59.0%) had the highest probability of being most effective; CON ranked lowest (15.5%). Thus, the depression network suggested that mind–body exercise and aerobic exercise were the most favorable modalities overall, whereas the relative position of resistance training was less certain. As shown in Table 1a, MBE (SMD = −1.34; 95% CI −2.30 to −0.39) and AE (SMD = −1.21; 95% CI −2.31 to −0.10) significantly outperformed CON in reducing depression. Given the low certainty of evidence, these findings should be interpreted as suggestive rather than definitive evidence for modality selection in depression.

**Table 1:** League table of outcomes in participants.

a. Depression					
MBE					
−0.13 (−1.59, 1.32)	AE				
−0.14 (−3.05, 2.78)	−0.00 (−2.75, 2.75)	RT			
−0.52 (−2.81, 1.77)	−0.39 (−2.59, 1.82)	−0.38 (−3.78,3.02)	HIIT		
−0.65 (−3.91, 2.62)	−0.51 (−3.82, 2.80)	−0.51 (−4.67, 3.66)	−0.12 (−3.88, 3.63)	CT	
**−1.34 (−2.30, −0.39)**	**−1.21 (−2.31, −0.10)**	−1.20 (−3.96, 1.55)	−0.82 (−2.90, 1.26)	−0.70 (−3.82, 2.43)	CON

b. Anxiety						
RT						
−0.20 (−1.98, 1.58)	TS					
−0.62 (−2.59, 1.35)	−0.42 (−1.65, 0.81)	CT				
−0.77 (−2.38, 0.83)	−0.57 (−1.52, 0.37)	−0.15 (−1.44, 1.13)	MBE			
−0.94 (−2.64, 0.75)	−0.74 (−1.88, 0.39)	−0.32 (−1.74, 1.09)	−0.17 (−1.00, 0.66)	AE		
−1.14 (−2.88, 0.59)	−0.94 (−2.14, 0.26)	−0.52 (−1.98, 0.94)	−0.37 (−1.28, 0.54)	−0.20 (−1.16, 0.76)	HIIT	
−1.45 (−2.99, 0.09)	**−1.25 (−2.14, −0.35)**	−0.83 (−2.05, 0.40)	**−0.67 (−1.11, −0.23)**	−0.50 (−1.20, 0.20)	−0.30 (−1.10, 0.49)	CON

c. Stress				
AE				
−0.20 (−1.69, 1.29)	TS			
−0.77 (−1.93, 0.38)	−0.57 (−1.64, 0.49)	MBE		
−0.77 (−1.83, 0.30)	−0.57 (−1.86, 0.73)	0.01 (−0.87, 0.89)	HIIT	
**−1.06 (−2.09, −0.03)**	−0.86 (−1.92, 0.21)	−0.28 (−0.78, 0.21)	−0.29 (−1.01, 0.43)	CON

Bold values indicate statistically significant results, with 95% CIs not crossing zero.

#### 3.2.2 Anxiety

Fourteen studies (*n* = 1,344) reported anxiety outcomes. Exercise significantly reduced anxiety compared with control (SMD = −0.69; 95% CI −1.00 to −0.38; *I*^2^ = 87.1%; Figure 2). This corresponded to a moderate standardized effect, with considerable heterogeneity across studies. Sensitivity analyses showed directionally consistent results, as presented in the anxiety-specific sensitivity analysis in Supplementary material, Section 6 (Figure 6.2). However, the certainty of evidence for anxiety remained very low, and the pooled estimate should therefore be interpreted cautiously.

Exploratory subgroup analyses revealed significant differences by intensity and intervention time. Exercise at 3.0–5.9 METs (SMD = −0.63; 95% CI −1.06 to −0.19) and ≥6 METs (SMD = −0.82; 95% CI −1.26 to −0.38) reduced anxiety significantly, whereas <3 METs did not (SMD = −0.54; 95% CI −1.46 to 0.38). Sessions of 30–59 min were effective (SMD = −0.74; 95% CI −1.05 to −0.43), whereas <30 min (SMD = −0.49; 95% CI −1.02 to 0.04) and ≥60 min (SMD = −0.82; 95% CI −2.97 to 1.33) were not significant. All subgroups defined by baseline depression, frequency and duration demonstrated significant improvements.

NMA geometry is shown in Figure 3b. Network *I*^2^ of 85.6% and *τ*^2^ of 0.35. SUCRA rankings (Figure 4b) indicated that RT (81.9%), TS (81.1%) and CT (57.4%) ranked highest, while CON ranked lowest (7.0%). However, rankings for TS and CT should be interpreted cautiously because these nodes were informed by few studies. Although RT and TS ranked highest, the league table showed clearer evidence for TS and MBE versus control, indicating that high ranking did not uniformly translate into statistically robust pairwise advantages.

In Table 1b, TS (SMD = −1.25; 95% CI −2.14 to −0.13) and MBE (SMD = −0.67; 95% CI −1.11 to −0.23) significantly reduced anxiety relative to CON. Overall, the anxiety network suggested potentially favorable rankings for RT and TS, while TS and MBE showed statistically significant comparisons with CON. Because the certainty of evidence for anxiety was very low, these modality-specific findings should be regarded as exploratory signals.

#### 3.2.3 Stress

Nine studies (*n* = 1,074) reported stress outcomes. Exercise significantly reduced stress compared with control (SMD = −0.45; 95% CI −0.73 to −0.16; *I*^2^ = 75.2%; Figure 2). This represented a small-to-moderate standardized effect, with substantial heterogeneity. Sensitivity analyses supported the robustness of the findings, with the stress-specific sensitivity analysis provided in Supplementary material, Section 6 (Figure 6.3), and the pooled effect remained directionally unchanged. After excluding Zhang et al. (36), the stress estimate also remained comparable with the primary analysis (SMD = −0.43; 95% CI −0.73 to −0.12; *I*^2^ = 77.7%; Supplementary Table S6.1), and the direction and statistical significance were unchanged.

Exploratory subgroup analyses showed significant differences for intensity, session duration and frequency. Exercise at 3.0–5.9 METs (SMD = −0.48; 95% CI −0.73 to −0.23) and ≥6 METs (SMD = −0.94; 95% CI −1.82 to −0.07) significantly reduced stress, whereas <3 METs did not (SMD = −0.15; 95% CI −0.73 to 0.44). Sessions of 30–59 min (SMD = −1.18; 95% CI −2.11 to −0.26) and ≥60 min (SMD = −0.58; 95% CI −1.00 to −0.16) were effective, whereas <30 min was not (SMD = −0.10; 95% CI −0.41 to 0.21). For frequency, interventions delivered 3–4 times per week (SMD = −0.46; 95% CI −0.86 to −0.05) and 5–7 times per week (SMD = −0.75; 95% CI −1.36 to −0.14) were significant, whereas 1–2 times per week was not (SMD = −0.42; 95% CI −0.86 to 0.01). All subgroups defined by baseline depression and duration showed significant reductions.

NMA structure is shown in Figure 3c. Network *I*^2^ of 71.4% and a *τ*^2^ of 0.17. SUCRA rankings (Figure 4c) indicated that AE (85.1%), TS (74.9%) and MBE (40.2%) were the most likely to reduce stress, while CON ranked lowest (10.4%). The ranking of TS should be interpreted cautiously given the limited number of contributing studies. In this network, aerobic exercise showed both the highest ranking probability and the clearest league-table evidence against control. As shown in Table 1c, AE significantly reduced stress compared with CON (SMD = −1.06; 95% CI −2.09 to −0.03). Taken together, these findings suggested AE as the most favorable comparative signal for stress. Because the certainty of evidence was low, this finding should be interpreted cautiously rather than as a definitive modality recommendation.

### 3.3 Risk of bias and publication bias

Among the 25 included RCTs, the overall risk of bias was judged as low in 15 studies and as raising some concerns in 10 studies; no study was rated as high risk overall. At the domain level, 24 studies were judged as low risk for the randomization process and 1 study raised some concerns; 20 studies were judged as low risk for deviations from intended interventions and 5 raised some concerns; 19 studies were judged as low risk for missing outcome data and 6 raised some concerns. All included studies were judged as low risk for measurement of the outcome and selection of the reported result. The domains that most frequently contributed to judgments of some concerns were deviations from intended interventions and missing outcome data. Concerns in the former domain were mainly related to the open-label nature of several behavioral exercise trials, in which participant blinding was not feasible and departures from assignment could not be fully excluded. Concerns regarding missing outcome data were mainly due to limited reporting of attrition or insufficient detail on missing-data handling. Full study-level, domain-specific RoB 2 judgments with supporting reasons for each domain-level decision are presented in Supplementary material, Section 4 (Supplementary Table S4), allowing transparent appraisal of the methodological quality of the included evidence.

Publication bias was assessed using funnel plots presented in Supplementary material, Section 5 (Figures 5.1–5.3), which show the outcome-specific distributions for depression, anxiety, and stress. Some asymmetry was observed across plots S5.1–S5.3. The corresponding Egger tests are also reported in Supplementary material, Section 5. Egger tests showed evidence of potential publication bias for anxiety (*p* < 0.05), suggesting that results for this outcome should be interpreted with caution. To further examine its impact, a trim-and-fill sensitivity analysis was conducted for the pairwise anxiety meta-analysis. The adjusted pooled effect was attenuated to SMD = −0.13 (95% CI −0.51 to 0.26), indicating that the original anxiety estimate may have been influenced by small-study effects or publication bias. Egger tests for depression and stress were >0.05, indicating no clear evidence of publication bias for these outcomes.

## 4 Discussion

This systematic review and network meta-analysis included 25 RCTs involving 2,127 university students and yielded three main findings. First, exercise was associated with improvements in depression, anxiety, and stress compared with control, with results remaining stable in sensitivity analyses, including analyses excluding the study with a routine physical education comparator. Second, the comparative evidence indicated outcome-specific patterns: for depression, MBE and AE were most consistently associated with favorable outcomes; for anxiety, MBE showed clearer associations, whereas TS and RT appeared favorable but were informed by limited evidence; for stress, AE was linked to the strongest comparative effect and ranked highest overall. Third, subgroup analyses suggested potentially relevant prescription-related patterns, with sessions of 30–59 min more frequently associated with benefits for depression and anxiety, whereas stress benefits were more often observed with sessions of 30 min or longer. Moderate to vigorous intensity appeared more relevant than light intensity for anxiety and stress, and stress outcomes were generally higher in studies with three or more sessions per week. These findings should be interpreted with caution due to heterogeneity, potential confounding, and the low to very low certainty of evidence. The observed outcome-specific patterns should therefore be viewed as tentative exploratory signals rather than direct guidance for symptom-targeted exercise selection.

Exercise may offer distinct value in university settings because it can be implemented as a low stigma, skills-based strategy that simultaneously targets sleep, fatigue, perceived competence, and day to day functioning, domains that often deteriorate during academic stress. The present findings indicate that exercise is associated with improvements across depression, anxiety, and stress, supporting its role as a broadly relevant option for student mental health rather than an intervention confined to a single symptom domain. This overall pattern aligns with previous syntheses in student and young adult populations showing that structured exercise or physical activity programmes can reduce symptoms of common mental health problems, while also highlighting that effect estimates may differ across reviews because of variation in baseline symptom severity, supervision and adherence, intervention content, and the diversity of outcome scales and comparators used (9, 10). Regular exercise can improve stress regulation and neurobiological function through changes in autonomic balance, hypothalamic pituitary adrenal axis responsivity, neurotrophic signaling, and inflammatory activity, all of which have been linked to depression, anxiety, and stress (41). At the behavioral level, exercise functions as behavioral activation and routine building, providing mastery experiences that strengthen self-efficacy and emotion regulation and may reduce rumination and threat focused attention through improved executive control (42). In parallel, participation in structured or group-based activities can enhance social connection and perceived support, which are particularly salient for students navigating transition and academic demands (43). These pathways provide plausible explanations for the observed associations between exercise and mental health outcomes in university students. However, given the certainty of the current evidence, integration into campus health promotion should be considered a promising direction that requires further confirmation rather than a definitive implementation recommendation.

Given the diversity of exercise programmes delivered in university settings, exploring whether specific modalities may relate differently to symptom domains is useful for generating future research priorities. The present NMA suggests that comparative effectiveness is outcome specific rather than uniform across depression, anxiety, and stress, which is consistent with earlier syntheses indicating that modality characteristics may influence mental health responses, although many previous reviews pooled heterogeneous interventions or focused on a single outcome, limiting direct modality comparisons (44). A plausible interpretation is that different modalities emphasize distinct therapeutic ingredients. Mind–body exercise may be well suited to depression and anxiety because it trains attentional control, interoceptive awareness, and emotion regulation skills, which can mitigate rumination and worry (45, 46). Aerobic exercise may be particularly relevant for perceived stress by improving physiological stress regulation and sleep related processes that amplify stress in students (47). Resistance training may contribute through mastery and self-efficacy gains and reductions in somatic tension, which can shift threat appraisal and avoidance (48). Team sports may preferentially affect anxiety through social connection and enjoyable, structured exposure to performance related arousal, potentially reducing isolation and strengthening perceived support (49). Concurrent training may integrate aerobic and strength pathways, but its benefits are likely sensitive to programme design and adherence. Overall, these findings suggest possible outcome specific differences across exercise modalities, but they should not be interpreted as definitive modality selection guidance. Comparative inferences remain tentative, particularly for anxiety and for sparsely represented modalities such as concurrent training and team sports.

Subgroup findings provide additional exploratory insight into how exercise characteristics may relate to university mental health, complementing the modality comparisons by highlighting modifiable programme features. Session duration appeared to be one relevant correlate across outcomes. For depression and anxiety, sessions of 30–59 min were more frequently associated with benefits, whereas very short sessions showed weaker or inconsistent effects and sessions of at least 60 min were not consistently beneficial. For stress, benefits were evident when sessions reached 30 min or longer, while sessions shorter than 30 min were not supported. These patterns are broadly in line with previous syntheses suggesting that moderate time efficient prescriptions may maximize psychological benefit while remaining feasible in student schedules, although direct comparisons across duration categories have been limited in earlier reviews (50, 51). The observed dose related signals may reflect a balance between physiological activation and behavioral feasibility. Sessions of around 30–59 min may provide sufficient volume to induce acute mood enhancing responses and repeated adaptations in sleep and stress regulation, while avoiding excessive perceived effort and time burden that can undermine adherence in students (52). In contrast, very short bouts may not accumulate enough exposure to shift stress appraisal or depressive symptoms, and longer sessions may increase fatigue and scheduling barriers, diluting net benefit through reduced participation (53). Intensity also appeared more informative for anxiety and stress, with moderate to vigorous activity associated with clearer effects than light intensity, which is consistent with the idea that a threshold of physiological arousal may be necessary to improve autonomic regulation and reduce somatic anxiety (54). Finally, the frequency signal for stress suggests that more regular exposure may be required to stabilize stress responses and reinforce coping routines. However, these subgroup patterns should be interpreted cautiously because they were derived from between-study comparisons and may be influenced by heterogeneity, residual confounding, limited power, and multiple testing. Accordingly, they are better viewed as hypothesis generating than as definitive causal thresholds for exercise prescription. This caution is especially relevant for anxiety, for which the certainty of evidence was very low and the pooled estimate was affected by potential publication bias.

This study has several strengths. By synthesizing evidence from RCTs in university students, it provides a coherent estimate of the overall association between exercise and improvements in depression, anxiety, and stress, supported by sensitivity analyses that enhance confidence in the stability of the findings. In addition, the combination of NMA with prespecified subgroup analyses extends prior work by moving beyond average effects to generate exploratory comparative evidence, including outcome specific modality patterns and prescription related signals that may help formulate hypotheses for future university based trials. These strengths should be interpreted alongside important limitations. The evidence base was methodologically heterogeneous, with many trials using open-label designs and a substantial proportion rated as having some concerns on RoB 2, while interventions were typically short term and adherence or completion reporting was often incomplete or inconsistently reported across studies. Adverse events were also rarely or unsystematically reported. This limits assessment of intervention fidelity, including the extent to which participants actually received the intended exercise dose, and constrains interpretation of feasibility and safety for practical implementation. Moreover, the network and data structure constrained precision for some comparisons, particularly for stress where fewer trials informed the network, and transitivity may have been challenged by between-trial differences in baseline symptom status, intervention delivery, and outcome measurement. One study used routine physical education as the comparator, which may have introduced some physical activity exposure into the control condition. Excluding this study did not materially change the pooled estimates for depression or stress, but this issue supports cautious interpretation of exercise-versus-control comparisons. Although exploratory network meta-regression analyses were performed to further examine potential effect modifiers, these models were based on single covariates and should be interpreted cautiously because several intervention nodes were sparse and some covariates were incompletely reported. In addition, some intervention nodes, particularly concurrent training, team sports, and resistance training, were informed by only a small number of studies, resulting in limited statistical power and reducing confidence in the stability and interpretability of the corresponding comparative rankings. Consistent with this, the certainty of evidence for the main network comparisons was low for depression and stress and very low for anxiety, indicating that comparative estimates, ranking patterns, and dose related findings should be interpreted as suggestive rather than definitive. Pairwise pooled effects also showed considerable heterogeneity for depression and anxiety and substantial heterogeneity for stress, indicating that the summary estimates should be interpreted as average effects across diverse trial settings rather than fixed treatment responses. Finally, the anxiety outcome showed evidence of potential publication bias, and the trim-and-fill sensitivity analysis substantially attenuated the pooled estimate, further reducing confidence in the precision of this finding. The restriction to English-language, peer-reviewed full-text articles may also have introduced both language bias and publication bias by excluding potentially relevant non-English studies and grey literature. This restriction, together with the geographic clustering of trials, may have affected the completeness of the evidence base and may limit the generalizability of the findings across university contexts.

## 5 Conclusion

This systematic review and network meta-analysis indicates that exercise interventions are associated with improvements in depression, anxiety, and stress in university students. The certainty of evidence was low for depression and stress and very low for anxiety, so the comparative findings should be interpreted cautiously. Mind–body and aerobic exercise showed the most consistent exploratory signals for depression, mind–body exercise was most clearly associated with improvements in anxiety, and aerobic exercise appeared most favorable for stress; however, these modality-specific patterns remain tentative. Prescription-related findings, including session duration, weekly frequency, and intensity, were derived from exploratory subgroup analyses and should be regarded as hypothesis-generating signals rather than prescriptive guidance. Overall, exercise may represent a promising component of university mental health promotion, but higher quality trials with standardized comparators, adequate sample sizes, and clearer reporting are required to further evaluate optimal modality and dose parameters.

## Data Availability

The original contributions presented in the study are included in the article/Supplementary material, further inquiries can be directed to the corresponding author.
