# Supplementary material for: Comparative effects of exercise modalities on depression, anxiety, and stress in university students: a systematic review and network meta-analysis of randomized controlled trials
Source: Front Public Health. 2026 May 29;14:1795525. doi: 10.3389/fpubh.2026.1795525 (PMC13259812; doi:10.3389/fpubh.2026.1795525)
Supplement: Supplementary file 1 [file Data_Sheet_1.docx]

**Supplementary**

Table of Contents:

[Supplementary 1: PRISMA Checklist 1](#_Toc534204563)

[Supplementary 2: Search Strategy 4](#_Toc1894445881)

[Supplementary 3: Characteristics of studies and subjects included in the review 5](#_Toc1347370016)

[Supplementary 4: Risk of Bias 1](#_Toc1262935083)1

[Supplementary 5: Publication bias 1](#_Toc431254745)3

[Supplementary 6: Sensitivity analysis 1](#_Toc1262935083)5

[Supplementary 7: Network meta-regression 2](#_Toc1262935083)2

[Supplementary 8: Global consistency test 2](#_Toc1262935083)3

[Supplementary 9: Results of the nodal splitting 25](#_Toc1347370016)

[Supplementary 10: [GRADE certainty for the network comparisons 27](#_Toc1347370016)](#_Toc1347370016)

**Supplementary 1: PRISMA Checklist**

| Item # | Checklist Item | Reported on Page # |
| --- | --- | --- |
|  |  | 1 |
| 1 | Identify the report as a systematic review incorporating a network meta-analysis (or related form of meta-analysis). | 1 |
| 2 | Provide a structured summary including, as applicable:  **Background:** main objectives  **Methods:** data sources; study eligibility criteria, participants, and interventions; study appraisal; and *synthesis methods, such as network meta-analysis.*  **Results:** number of studies and participants identified; summary estimates with corresponding confidence/credible intervals; treatment rankings may also be discussed. Authors may choose to summarize pairwise comparisons against a chosen treatment included in their analyses for brevity.  **Discussion/Conclusions:** limitations; conclusions and implications of findings.  **Other:** primary source of funding; systematic review registration number with registry name. | 1-2 |
| 3 | Describe the rationale for the review in the context of what is already known*, including mention of why a meta-analysis of randomized controlled trials and dose-response analysis has been conducted.* | 3-5 |
| 4 | Provide an explicit statement of questions being addressed, with reference to participants, interventions, comparisons, outcomes, and study design (PICOS). | 5-6 |
| 5 | Indicate whether a review protocol exists and if and where it can be accessed (e.g., Web address); and, if available, provide registration information, including registration number. | 5 |
| 6 | Specify study characteristics (e.g., PICOS, length of follow-up) and report characteristics (e.g., years considered, language, publication status) used as criteria for eligibility, giving rationale. *Clearly describe eligible treatments included in the treatment network, and note whether any have been clustered or merged into the same node (with justification).* | 28-29 |
| 7 | Describe all information sources (e.g., databases with dates of coverage, contact with study authors to identify additional studies) in the search and date last searched. | 5 |
| 8 | Present full electronic search strategy for at least one database, including any limits used, such that it could be repeated. | Supplementary 2 |
| 9 | State the process for selecting studies (i.e., screening, eligibility, included in systematic review, and, if applicable, included in the meta-analysis). | 5-6 |
| 10 | Describe method of data extraction from reports (e.g., piloted forms, independently, in duplicate) and any processes for obtaining and confirming data from investigators. | 6-7 |
| 11 | List and define all variables for which data were sought (e.g., PICOS, funding sources) and any assumptions and simplifications made. | 7-8 |
| S1 | Describe methods used to explore the geometry of the treatment network under study and potential biases related to it. This should include how the evidence base has been graphically summarized for presentation, and what characteristics were compiled and used to describe the evidence base to readers. | 8 |
| 12 | Describe methods used for assessing risk of bias of individual studies (including specification of whether this was done at the study or outcome level), and how this information is to be used in any data synthesis. | 8, Supplementary 4 |
| 13 | State the principal summary measures (e.g., risk ratio, difference in means). Also describe the use of additional summary measures assessed, such as treatment rankings and surface under the cumulative ranking curve (SUCRA) values, as well as modified approaches used to present summary findings from meta-analyses. | 8-10 |
| 14 | Describe the methods of handling data and combining results of studies for each network meta-analysis. This should include, but not be limited to:   - Handling of multi-arm trials; - Selection of variance structure; - Selection of prior distributions in Bayesian analyses; and - Assessment of model fit. | 8-10 |
| S2 | Describe the statistical methods used to evaluate the agreement of direct and indirect evidence in the treatment network(s) studied. Describe efforts taken to address its presence when found. | Supplementary 7 |
| 15 | Specify any assessment of risk of bias that may affect the cumulative evidence (e.g., publication bias, selective reporting within studies). | 9,  Supplementary 6 |
| 16 | Describe methods of additional analyses if done, indicating which were pre-specified. This may include, but not be limited to, the following:   - Sensitivity or subgroup analyses; - Meta-regression analyses; - Alternative formulations of the treatment network; and - Use of alternative prior distributions for Bayesian analyses (if applicable). | 8-10 |
| 17 | Give numbers of studies screened, assessed for eligibility, and included in the review, with reasons for exclusions at each stage, ideally with a flow diagram. | Figure 1 |
| S3 | Provide a network graph of the included studies to enable visualization of the geometry of the treatment network. | Figure 5 |
| S4 | Provide a brief overview of characteristics of the treatment network. This may include commentary on the abundance of trials and randomized patients for the different interventions and pairwise comparisons in the network, gaps of evidence in the treatment network, and potential biases reflected by the network structure. | Supplementary 9 |
| 18 | For each study, present characteristics for which data were extracted (e.g., study size, PICOS, follow-up period) and provide the citations. | Supplementary 3 |
| 19 | Present data on risk of bias of each study and, if available, any outcome level assessment. | 11,  Supplementary 4, and Supplementary 6 |
| 20 | For all outcomes considered (benefits or harms), present, for each study: 1) simple summary data for each intervention group, and 2) effect estimates and confidence intervals. *Modified approaches may be needed to deal with information from larger networks.* | Supplementary 3 |
| 21 | Present results of each meta-analysis done, including confidence/credible intervals. In larger networks, authors may focus on comparisons versus a particular comparator (e.g. placebo or standard care), with full findings presented in an appendix. League tables and forest plots may be considered to summarize pairwise comparisons. If additional summary measures were explored (such as treatment rankings), these should also be presented. | Figure 2, Figure 3, and Figure 4 |
| S5 | Describe results from investigations of inconsistency. This may include such information as measures of model fit to compare consistency and inconsistency models, *P* values from statistical tests, or summary of inconsistency estimates from different parts of the treatment network. | Supplementary 8, and Supplementary 9 |
| 22 | Present results of any assessment of risk of bias across studies for the evidence base being studied. | 11,  Supplementary 4, and Supplementary 6 |
| 23 | Give results of additional analyses, if done (e.g., sensitivity or subgroup analyses, meta-regression analyses*, alternative network geometries studied, alternative choice of prior distributions for Bayesian analyses,* and so forth). | Figure 4,  and Figure 5 |
| 24 | Summarize the main findings, including the strength of evidence for each main outcome; consider their relevance to key groups (e.g., healthcare providers, users, and policy-makers). | 14-19 |
| 25 | Discuss limitations at study and outcome level (e.g., risk of bias), and at review level (e.g., incomplete retrieval of identified research, reporting bias). *Comment on the validity of the assumptions, such as transitivity and consistency. Comment on any concerns regarding network geometry (e.g., avoidance of certain comparisons).* | 19 |
| 26 | Provide a general interpretation of the results in the context of other evidence, and implications for future research. | 19 |
| 27 | Describe sources of funding for the systematic review and other support (e.g., supply of data); role of funders for the systematic review. This should also include information regarding whether funding has been received from manufacturers of treatments in the network and/or whether some of the authors are content experts with professional conflicts of interest that could affect use of treatments in the network. | 19 |

PICOS = population, intervention, comparators, outcomes, study design.

**Supplementary 2: Search Strategy**

## Database: PubMed <inception to October 31 2025>

***Search Strategy:***

( "Students, College"[Mesh] OR "Students"[Mesh] OR "universities"[tiab] OR university[tiab] OR university student*"[tiab] OR "college"[tiab] OR "college student*"[tiab] OR undergraduate*[tiab] OR "higher education"[tiab] OR "tertiary education"[tiab] OR campus[tiab] OR student*[tiab])

AND

( "Exercise"[Mesh] OR "Motor Activity"[Mesh] OR "Physical Fitness"[Mesh] OR "Sports"[Mesh] OR exercise*[tiab] OR "physical activity"[tiab] OR "physical activities"[tiab] OR "aerobic exercise*"[tiab] OR aerobic[tiab] OR "endurance training"[tiab] OR "endurance exercise*"[tiab] OR "resistance training"[tiab] OR "strength training"[tiab] OR "strength exercise*"[tiab] OR "weight training"[tiab] OR "concurrent training"[tiab] OR "combined training"[tiab] OR "interval training"[tiab] OR "high intensity interval"[tiab] OR HIIT[tiab] OR "mind-body"[tiab] OR "mind body"[tiab] OR yoga[tiab] OR "tai chi"[tiab] OR qigong[tiab] OR pilates[tiab] OR "team sport*"[tiab] OR football[tiab] OR soccer[tiab] OR basketball[tiab] OR volleyball[tiab] OR handball[tiab] OR rugby[tiab] OR "small-sided game*"[tiab] OR "physical education"[tiab] OR "exercise training"[tiab])

AND

( "Depression"[Mesh] OR "Depressive Disorder"[Mesh] OR "Anxiety"[Mesh] OR "Anxiety Disorders"[Mesh] OR "Stress, Psychological"[Mesh] OR "Stress, Psychological/psychology"[Mesh] OR "Adaptation, Psychological"[Mesh] OR depression[tiab] OR depressive[tiab] OR depressed[tiab] OR melancholia[tiab] OR dysphoria[tiab] OR "depressive symptom*"[tiab] OR "emotional depression"[tiab] OR anxiety[tiab] OR anxious[tiab] OR "anxiety symptom*"[tiab] OR stress[tiab] OR "psychological stress"[tiab] OR "mental stress"[tiab] OR distress[tiab] OR "psychological distress"[tiab] OR BDI[tiab] OR "Beck Depression Inventory"[tiab] OR "BDI-II"[tiab] OR "Center for Epidemiologic Studies Depression"[tiab] OR CES-D[tiab] OR CESD-R[tiab] OR "Self-Rating Depression Scale"[tiab] OR SDS[tiab] OR "Zung Self-Rating Depression Scale"[tiab] OR "Patient Health Questionnaire"[tiab] OR PHQ-9[tiab] OR PHQ-4[tiab] OR "Hospital Anxiety and Depression Scale"[tiab] OR HADS[tiab] OR "HADS-D"[tiab] OR "HADS-A"[tiab] OR "Depression Anxiety Stress Scale"[tiab] OR DASS-21[tiab] OR "State-Trait Anxiety Inventory"[tiab] OR STAI[tiab] OR "STAI 6"[tiab] OR SAI[tiab] OR "Self-Rating Anxiety Scale"[tiab] OR SAS[tiab] OR BAI[tiab] OR GAD-7[tiab] OR HAMA[tiab] OR CPSS[tiab] OR "Perceived Stress Scale"[tiab] OR PSS[tiab] OR "PSS-10"[tiab] OR "DASS-21 Stress"[tiab])

AND

( randomized controlled trial[pt] OR controlled clinical trial[pt] OR randomized[tiab] OR randomised[tiab] OR randomly[tiab] OR placebo[tiab] OR trial[ti] OR "clinical trials as topic"[Mesh:noexp]) NOT ( animals[mh] NOT (humans[mh] AND animals[mh]))

# Supplementary 3: Characteristics of studies and subjects included in the review

| **Study** | **Country/Region** | **Study design** | **Subjects**  **(intervention/ control)** | **Sex (male/female)**  **(intervention/ control)** | **Mean age**  **(intervention/ control)** | **BMI**  **(intervention/ control)** | **Depression** | **Baseline** | **Cut off** | **Intervention detail** | | **Intensity** | **METs** | **Session duration** | **Training frequency** | **Duration** | **Completion rate** | **Outcomes** |
| --- | --- | --- | --- | --- | --- | --- | --- | --- | --- | --- | --- | --- | --- | --- | --- | --- | --- | --- |
|  |  |  |  |  |  |  |  |  |  | **Intervention group** | **Control group** |  |  |  |  |  |  |  |
| Ji et al. (2022) | China | open-label RCT | 197 (66/64/67) | 35/31 vs. 39/25 vs. 41/26 | 21.42 ± 2.4 vs. 22.31 ± 3.1 vs. 21.90 ± 2.1 | 27.43 ± 5.5 vs. 27.82 ± 4.9 vs. 27.50 ± 5.0 | N/A | N/A | N/A | (a) Basketball game (including 10 minutes of warm-up, 40 minutes of game, and 10 minutes of cool-down and discussion) (b) Running + Strength Training Combination | No exercise intervention | NA | (a) 8 (b) 5.7 | 40 min | 2 × per week | 6 weeks | 66/78 vs. 67/79 | Anxiety |
| Eather et al. (2018) | Australia | open-label RCT | 53 (26/27) | 8/14 vs. 10/21 | 20.23 ± 1.72 vs. 20.48 ± 2.01 | 24.11 ± 4.06 vs. 23.10 ± 4.20 | N/A | N/A | N/A | Multimodal HIIT: Each work interval consists of a combination of aerobic exercise and core/bodyweight resistance exercise. | Wait-list control | ≥85% HRmax | 8 | 10 min | 3 × per week | 8 weeks | 75.50% | Anxiety, Stress |
| Xiao et al. (2021) | China | open-label RCT | 96 (31/31/34) | 23/8 vs. 24/7 vs. 24/10 | 18.95 ± 0.89 vs. 19.21 ± 1.02 vs. 19.71 ± 1.77 | N/A | N/A | N/A | N/A | (a) Team basketball training + games (half-court 3v3/2v2, including basic movements, techniques and confrontation). (b) Eight-Section Brocade Qigong (a complete set of 8 movements, including the starting and ending postures) | No exercise intervention, maintaining the original lifestyle | Heart rate approximately 120–150 beats per minute | (a) 8 (b) 3 | 70 min | 3 × per week | 12 weeks | 31/33 vs. 31/33 vs. 34/34 | Anxiety, Stress |
| Zhang et al. (2022) | China | open-label RCT | 46 (23/23) | 18/14 vs. 20/12 | 19.39 ± 1.73 vs. 19.61 ± 1.19 | N/A | CES-D | 26.96 ± 4.80 vs. 26.78 ± 5.63 | ≥16 | Jogging, basketball, and outreach training | Irregular exercise/sedentary lifestyle, maintain your original lifestyle | 60%–65% HRmax | 6 | 40 min | 3 × per week | 12 weeks | N/A | Depression, Sleep |
| Falsafi (2016) | USA | open-label RCT | 44 (21/23) | 3/18 vs. 3/20 | 22.1 ± 1.5 | N/A | BDI | 20.0 ± 10.6 vs. 20.2 ± 9.1 | ≥14 | Gentle Hatha Yoga | No exercise intervention | NA | 2.5 | 30 min | 5 × per week | 8 weeks | 67/84 | Depression, Anxiety, Stress |
| Zhang et al. (2023a) | China | single-blind RCT | 18 (9/9) | 2/7 vs. 3/6 | 24.20 ± 4.07 vs. 22.50 ± 5.95 | 21.92 vs. 20.96 | SDS | 69.29 ± 12.16 vs. 57.50 ± 11.32 | ≥53 | Bafa Wubu of Tai Chi | No exercise intervention, maintaining normal daily life | NA | 3 | 40 min | 5 × per week | 8 weeks | N/A | Depression, Anxiety |
| Philippot et al. (2022) | Belgium | single-blind RCT | 28 (13/15) | 2/11 vs. 1/14 | 20.69 ± 1.44 vs. 20.93 ± 1.94 | 22.67 ± 4.21 vs. 21.35 ± 3.01 | GAD-7 | 16.8 ± 9.6 vs. 16.9 ± 9.2 | ≥ 5 | HIIT with online video instruction. A combination of bodyweight cardio and muscle strength training (such as squats, burpees, mountain climbers, planks, push-ups, etc.). | No exercise intervention | HRmax ≥80% | 8 | 10 min | 3 × per week | 4 weeks | 13/15 vs. 15/15 | Depression, Anxiety, Stress |
| Zhao et al. (2023) | China | single-blind RCT | 86 (29/29/28) | 8/21 vs. 9/20 vs. 8/20 | 20.72 ± 2.05 vs. 21.66 ± 1.97 vs. 21.21 ± 2.25 | N/A | SDS | 62.55 ± 6.44 vs. 64.48 ± 5.72 vs. 60.93 ± 6.81 | ≥53 | (a) brisk walking + jogging on the treadmill (b) Dumbbell + Resistance Band Resistance Training | No exercise intervention, maintain the original lifestyle | Initial: 50–60% HRR Intermediate: 60–75% HRR Advanced: 75–90% HRR | (a) 5.7 (b) 4.1 | (a) 30 min (b) 35 min | 3 × per week | 12 weeks | 29/29 vs. 29/29 vs. 28/28 | Depression |
| Li et al. (2022) | China | open-label RCT | 27 (13/14) | 0/13 vs. 0/14 | 22.6 ± 2.5 vs. 22.5 ± 2.0 | 21.8 ± 2.7 vs. 20.7 ± 2.0 | N/A | N/A | N/A | Cluster training includes three exercises: barbell bench press (pectoralis major), lat pulldown (latissimus dorsi), and seated leg extension (quadriceps). | Unstructured movement intervention to maintain daily life habits | 70% 1RM | 6 | 40 min | 2 × per week | 8 weeks | 13/13 vs. 14/14 | Anxiety |
| Ghorbani et al. (2014) | Iran | open-label RCT | 30 (15/15) | 0/15 vs. 0/15 | 26.06 ± 1.18 vs. 26.33 ± 1.30 | 22.37 ± 1.60 vs. 22.01 ± 1.86 | GHQ-28 Depression | 4.00 ± 1.80 vs. 5.33 ± 5.20 | ≥ 4 | Running + Rope Skipping | No training, maintaining the original lifestyle | NA | 9.6 | 40 min | 3 × per week | 6 weeks | 15/15 vs. 15/15 | Depression, Anxiety |
| Kim et al. (2004) | Korea | single-blind RCT | 54 (26/28) | 0/26 vs. 0/28 | 19-21 years | N/A | DSI | 57.78 ± 0.43 vs. 59.50 ± 0.47 | ≥50 | Meridian exercise (a type of Eastern Qigong exercise) | No exercise intervention, maintain daily life | NA | 3 | 30 min | 2 × per week | 6 weeks | 26/28 | Depression, Anxiety |
| López-Rodríguez et al. (2017) | Spain | open-label RCT | 95 (42/53) | 10/32 vs. 14/39 | 22.45 ± 4.96 vs. 21.77 ± 4.53 | N/A | CES-D | 24.17 ± 12.71 vs. 19.81 ± 10.12 | ≥16 | Biodanza (therapeutic dance) includes a warm-up (low-intensity exercise), an activation exercise (walking, dancing, or working out), and a celebratory segment. | No exercise intervention, maintain daily life | NA | 4.5 | 40 min | 1 × per week | 4 weeks | 42/52 vs. 53/61 | Depression, Stress |
| Zhang et al. (2023b) | China | single-blind RCT | 73 (34/39) | 0/34 vs. 0/39 | 19.23 ± 0.98 vs. 19.16 ± 1.05 | 20.71 ± 3.43 vs. 21.03 ± 3.87 | SCL-90 | 0.86 ± 0.48 vs. 0.87 ± 0.51 | N/A | Eight-Section Brocade Qigong (a complete set of 8 movements, including the starting and ending postures) | No exercise intervention, maintain daily life | NA | 3 | 40 min | 3 × per week | 12 weeks | 34/39 vs. 39/39 | Depression, Anxiety |
| Chang et al. (2022) | USA | open-label RCT | 679 (327/352) | 88/239 vs. 92/260 | 21.4 ± 4.7 vs. 21.3 ± 5.0 | N/A | PHQ-4 | 4.16 ± 1.78 vs. 4.31 ± 1.82 | ≥ 3 | Online Isha Upa Yoga, with pre-recorded video instruction. It includes two practices: Yoga Namaskar (posture sequence and breathing) and Nadi Shuddhi (alternating nostril breathing). | Waiting list | NA | 2.5 | 15 min | 3 × per week | 4 weeks | 212/326 vs. 193/352 | Depression, Anxiety, Stress |
| Paolucci et al. (2018) | Canada | open-label RCT | 61 (20/19/22) | 5/13 vs. 6/13 vs. 5/13 | 21 ± 2 | 21.7 ± 0.5 | BDI-II | 13.2 ± 9.5 vs. 11.4 ± 8.8 vs. 16.7 ± 9.5 | ≥14 | (a) HIIT (stationary cycle ergometer): 10×60 s high-intensity intermittent pulses, 80% maximum power (max wattage);  10×60 s active recovery, 30% maximum power (≥50W); Average HR 174±2 bpm; RPE 15±0.3/20 (b) Power bike moderate-intensity continuous riding | No training, maintaining a sedentary lifestyle | NA | (b) 8.8 (b) 4.8 | (a) 20 min (b) 30 min | 3 × per week | 6 weeks | 18/20 vs. 19/19 vs. 18/22 | Depression, Anxiety, Stress |
| Papp et al. (2019) | Sweden | single-blind RCT | 44 (21/23) | 3/18 vs. 3/20 | 25 ± 3.2 vs. 25 ± 2.7 | N/A | HADS | 4.0 ± 3.0 vs. 4.4 ± 3.6 | ≥11 | High intensity hatha yoga | No yoga/No home workouts/No changes to existing exercise habits | NA | 4 | 60 min | 1 × per week | 6 weeks | N/A | Depression, Anxiety, Stress |
| Roth et al. (1987) | USA | open-label RCT | 44 (23/21) | 9/9 vs. 8/10 | 18.9 ± 1.3 | N/A | BDI | 7.8 ± 6.4 vs. 7.0 ± 6.9 | ≥14 | Running + brisk walking when necessary | There was no training involved; it only occurred during measurement. | ≥75% HRmax | 5.7 | 30 min | 3 × per week | 11 weeks | N/A | Depression, Anxiety |
| Saltan et al. (2020) | Turkey | single-blind RCT | 64 (29/35) | 6/23 vs. 4/31 | 18.82 ± 1.07 vs. 19.42 ± 1.38 | 22.11 ± 3.71 vs. 20.84 ± 2.30 | BDI | 9.07 ± 5.45 vs. 11.63 ± 4.94 | ≥14 | Mat-work Pilates：hundred/half roll-up/single-leg，stretch/crisscross/spine stretch，forward/swan/swimming/saw/bridge/side-leg lifts | Irregular exercise/no training | NA | 3 | 45 min | 3 × per week | 12 weeks | 29/35 vs. 35/35 | Depression |
| Smits et al. (2008) | USA | open-label RCT | 39 (19/20) | 2/17 vs. 7/13 | 19.53 ± 0.35 vs. 22.65 ± 2.09 | N/A | BDI | 13.16 ± 7.98 vs. 13.43 ± 6.21 | ≥14 | Treadmill aerobic training can help you achieve your target heart rate by brisk walking or jogging. | Wait-list control | 70% HRmax | 7 | 20 min | 3 × per week | 2 weeks | 16/19 vs. 18/19 | Depression, Anxiety |
| Abic et al. (2024) | North Cyprus | open-label RCT | 34 (17/17) | 0/17 vs. 0/17 | 21.93 ± 1.90 | 21.93 ± 1.90 vs. 19.91 ± 2.38 | DASS-21 | 17.24 ± 4.92 vs. 14.00 ± 4.53 | ≥10 | Yoga is taught by certified researchers. | No exercise intervention | NA | 2.5 | 20 min | 3 × per week | 8 weeks | 17/17 vs. 17/17 | Depression, Anxiety, Stress |
| Wang et al. (2024) | China | open-label RCT | 93 (46/47) | 24/22 vs. 25/22 | 19.21±0.17 vs. 19.18±0.32 | N/A | SCL-90-R | 2.22 ± 1.05 vs. 2.23 ± 0.87 | ≥ 2 | Health Qigong Baduanjin | No exercise intervention, maintain daily life | NA | 3 | 50 min | 3 × per week | 16 weeks | N/A | Depression, Anxiety |
| Wu et al. (2023) | China | single-blind RCT | 103 (49/54) | 15/34 vs. 18/36 | 19.33 ± 0.74 vs. 19.00 ± 1.48 | 20.75 ± 2.67 vs. 20.95 ± 4.40 | CES-D | 20.95 ± 4.40 vs. 22.00 ± 8.15 | ≥16 | 24-Form Simplified Tai Chi | Wait-list control | NA | 3 | 45 min | 3 × per week | 12 weeks | 103/112 | Depression, Anxiety |
| Zhang et al. (2018) | China | single-blind RCT | 62 (32/30) | 21/41 | 18.41±2.01 | N/A | PHQ-9 | 8.66±2.12 vs. 8.23±2.11 | ≥5 | Mindfulness Tai Chi | Regular physical education courses | NA | 3 | 90 min | 2 × per week | 8 weeks | 30/32 vs. 32/32 | Depression, Stress |
| Zieff et al. (2022) | USA | open-label RCT | 27 (18/9) | 3/15 vs. 2/7 | 20.91±2.91 vs. 20.06±0.95 | 22.64±2.83 vs. 27.28±6.8 | DASS-21 | 24.97±2.13 | ≥5 | Cycle ergometer or treadmill | No exercise intervention | ≥40% HRR | 6 | 40 min | 3 × per week | 4 weeks | 16/18 vs. 6/9 | Depression, Stress |
| Sadeghi et al. (2016) | Sweden | single-blind RCT | 30 (16/14) | 13/3 vs. 11/3 | 20.93 ± 1.06 vs. 20.92 ± 1.20 | N/A | BDI-II | 23.12 ± 3.61 vs. 22.70 ± 4.84 | ≥14 | Aerobic exercise/Aerobic-style aerobic training | No exercise intervention | 60%–80% HR | 7.3 | 35 min | 1-2 × per week | 8 weeks | NA | Depression |

N/A, not available; RCT, randomized controlled trial; BMI, body mass index; MET, metabolic equivalent of task; HR, heart rate; HRmax, maximum heart rate; HRR, heart rate reserve; bpm, beats per minute; 1RM, one-repetition maximum; RPE, rating of perceived exertion; HIIT, high-intensity interval training; VO₂max, maximal oxygen uptake; QoL, quality of life; HRQoL, health-related quality of life; ACSM, American College of Sports Medicine; BDI, Beck Depression Inventory; BDI-II, Beck Depression Inventory-II; CES-D, Center for Epidemiologic Studies Depression Scale; CESD-R, Center for Epidemiologic Studies Depression Scale–Revised; SDS, Self-Rating Depression Scale; DSI, Depression Status Inventory; GHQ-28, General Health Questionnaire-28; HADS, Hospital Anxiety and Depression Scale; GAD-7, Generalized Anxiety Disorder-7; BAI, Beck Anxiety Inventory; HAMA, Hamilton Anxiety Rating Scale; PHQ-4, 4-item Patient Health Questionnaire; PHQ-9, 9-item Patient Health Questionnaire; SCL-90, Symptom Checklist-90; SCL-90-R, Symptom Checklist-90-Revised; DASS-21, 21-item Depression Anxiety Stress Scale; CPSS, Chinese Perceived Stress Scale; PSS, Perceived Stress Scale; SAI, State Anxiety Inventory; STAI, State-Trait Anxiety Inventory; SAS, Self-Rating Anxiety Scale; HDRS, Hamilton Depression Rating Scale.

# Supplementary 4: Risk of Bias

| **Study** | **Randomization process** | **Deviations from intended interventions** | **Missing outcome data** | **Measurement of the outcome** | **Selection of the reported result** | **Overall bias** |
| --- | --- | --- | --- | --- | --- | --- |
| Ji et al. (2022) | Low: Random allocation was reported, and no important baseline imbalance suggesting failure of randomization was evident. | Some concerns: This was an open-label behavioral trial, and lack of participant blinding may have allowed deviations from intended intervention or contamination between groups. | Low: Outcome data appeared sufficiently complete for the main analysis. | Low: Anxiety outcomes were measured using the same procedures across groups. | Low: No clear evidence of selective reporting was identified from the available report. | Some concerns |
| Eather et al. (2018) | Low: Randomization appeared appropriate, and baseline characteristics were broadly comparable. | Low: The intervention and wait-list control were clearly separated, with no major deviations likely to affect the estimate. | Low: Attrition did not appear sufficient to materially bias results. | Low: Outcomes were assessed consistently across groups using the same methods. | Low: No signal of selective outcome reporting was evident. | Low |
| Xiao et al. (2021) | Low: Random allocation was reported, and no obvious indication of compromised sequence generation was evident. | Low: Intervention delivery and the no-exercise control were sufficiently distinct, with no major concerns about departures from assignment. | Low: Completion was high and balanced across arms. | Low: Mental health outcomes were measured similarly across all groups. | Low: Reported outcomes were consistent with study objectives, and no selective reporting signal was evident. | Low |
| Zhang et al. (2022) | Low: Randomization was reported, and baseline CES-D scores were comparable. | Low: No important deviations from intended intervention were apparent. | Low: No material concern regarding completeness of outcome data was evident. | Low: The same validated outcome assessment was used in both groups. | Low: No evidence of selective reporting was identified. | Low |
| Falsafi (2016) | Low: Randomization appeared acceptable, and baseline depression scores were similar between groups. | Low: No major concern was identified regarding departures from assigned intervention. | Some concerns: Retention and completion were not described in sufficient detail to exclude possible attrition-related bias. | Low: Standardized self-report measures were used consistently across groups. | Low: No clear indication of selective reporting was observed. | Some concerns |
| Zhang et al. (2023a) | Low: Random assignment was reported, and no major baseline imbalance was evident. | Low: Participants appeared to receive the intended intervention and control as planned. | Low: No important concern regarding missing outcome data was identified. | Low: Validated scales were used with the same assessment approach in both groups. | Low: No signal of selective reporting was evident. | Low |
| Philippot et al. (2022) | Low: Randomization appeared acceptable, and baseline GAD-7 values were comparable. | Some concerns: The online exercise format and lack of participant blinding may have permitted deviations from the intended regimen. | Low: Outcome data appeared sufficiently complete for analysis. | Low: The same validated outcome measures were used across groups. | Low: No evidence of selective reporting was identified. | Some concerns |
| Zhao et al. (2023) | Low: Randomization was reported, and baseline SDS scores were reasonably balanced across groups. | Low: No major concern was identified regarding departures from assigned intervention. | Low: Outcome data appeared complete across arms. | Low: Measurement procedures were consistent across groups. | Low: Outcomes reported were aligned with the study objectives, and no selective reporting signal was apparent. | Low |
| Li et al. (2022) | Low: Randomization appeared adequate, and no substantial baseline imbalance was evident. | Some concerns: This was a behavioral exercise trial without participant blinding; adherence-related deviations or contamination could not be fully excluded. | Some concerns: Handling of missing data and the analysis population were not described in enough detail to fully rule out attrition-related bias. | Low: Anxiety outcomes were assessed similarly in both groups. | Low: No clear indication of selective reporting was present in the available report. | Some concerns |
| Ghorbani et al. (2014) | Low: Random allocation was reported, and groups were broadly comparable at baseline. | Low: No important deviation from intended intervention was apparent. | Low: Outcome data appeared complete and balanced. | Low: The same validated instrument was applied across groups. | Low: Reported outcomes did not suggest selective reporting. | Low |
| Kim et al. (2004) | Low: Randomization appeared acceptable, and there was no clear sign of baseline imbalance indicating a problem with allocation. | Low: Participants appeared to follow intended group assignment without major concern. | Low: No substantial issue with missing outcome data was apparent. | Low: Outcome measurement was standardized across groups. | Low: No evidence of selective reporting was identified. | Low |
| López-Rodríguez et al. (2017) | Low: Randomization appeared acceptable, and baseline values were not clearly incompatible with successful allocation. | Low: No major concern was identified regarding deviations from intended intervention. | Some concerns: Completion was not fully balanced, and the potential impact of missing data could not be excluded with confidence. | Low: The same outcome measures were applied across groups. | Low: No obvious evidence of selective reporting was identified. | Some concerns |
| Zhang et al. (2023b) | Low: Randomization was reported, and baseline SCL-90 values were comparable. | Low: Intervention implementation appeared consistent with assignment. | Low: Outcome data appeared sufficiently complete for analysis. | Low: Outcomes were measured using the same procedures in both groups. | Low: No selective reporting signal was apparent. | Low |
| Chang et al. (2022) | Low: Randomization appeared appropriate, and baseline PHQ-4 scores were similar across groups. | Low: Although open-label, no major protocol deviations likely to affect the estimate were apparent. | Low: Attrition was reported and did not appear sufficient to materially bias the findings. | Low: Validated outcomes were measured consistently across groups. | Low: Reported outcomes were consistent with the stated design, and no clear selective reporting signal was evident. | Low |
| Paolucci et al. (2018) | Low: Randomization appeared adequate, and baseline BDI-II values were reasonably comparable across groups. | Low: Intervention adherence and group distinction appeared acceptable, with no major concern about deviations from assignment. | Low: Outcome data were sufficiently complete for the main comparisons. | Low: Outcomes were measured consistently across study arms. | Low: No evidence of selective non-reporting was identified. | Low |
| Papp et al. (2019) | Low: Random assignment appeared acceptable, and baseline HADS values were broadly comparable. | Low: No major concern was identified regarding deviations from intended intervention. | Low: Missing data were not judged sufficient to introduce material bias. | Low: Outcomes were measured using standardized instruments across groups. | Low: No clear evidence of selective reporting was found. | Low |
| Roth et al. (1987) | Low: No clear evidence suggested failure of randomization, and baseline depression values were broadly similar. | Low: No major concern was identified regarding deviations from intended assignment. | Some concerns: Follow-up completeness and handling of missing data were insufficiently reported to confidently exclude attrition-related bias. | Low: Outcome assessment methods appeared comparable between groups. | Low: No obvious indication of selective reporting was present in the available report. | Some concerns |
| Saltan et al. (2020) | Low: Randomization appeared acceptable, and baseline depression scores were reasonably balanced. | Low: No major concern was identified regarding departures from intended intervention. | Low: Outcome data appeared largely complete and balanced between groups. | Low: The same validated outcome assessment was used in both groups. | Low: No clear evidence of selective outcome reporting was identified. | Low |
| Smits et al. (2008) | Some concerns: Randomization was reported, but insufficient detail on sequence generation or allocation concealment left some uncertainty about the randomization process. | Low: No major deviation from the assigned intervention likely to substantially influence the effect estimate was evident. | Some concerns: Some attrition occurred, and the handling of missing data was not described in enough detail to exclude bias with confidence. | Low: Outcome measurement was standardized and applied similarly across groups. | Low: No signal of selective reporting was evident from the available paper. | Some concerns |
| Abic et al. (2024) | Low: Randomization appeared acceptable, and baseline DASS-21 values were broadly comparable. | Low: No important concern was identified regarding deviations from intended intervention. | Some concerns: Reporting on the analysis population and missing-data handling was limited, leaving some uncertainty. | Low: Validated outcome measures were applied consistently across groups. | Low: No clear evidence of selective reporting was identified. | Some concerns |
| Wang et al. (2024) | Low: Random assignment appeared acceptable, and baseline values were comparable between groups. | Low: No important deviation from intended intervention was evident. | Low: No substantial concern regarding missing outcome data was apparent. | Low: The same outcome measurement approach was used across groups. | Low: No clear selective reporting signal was observed. | Low |
| Wu et al. (2023) | Low: Randomization appeared appropriate, and baseline CES-D values were similar between groups. | Low: Intervention and wait-list control conditions were sufficiently distinct, with no major concern about deviations from assignment. | Low: Completion appeared acceptable and unlikely to materially bias the results. | Low: Standardized outcome measures were applied similarly across groups. | Low: No evidence of selective outcome reporting was identified. | Low |
| Zhang et al. (2018) | Low: No indication of failed randomization was evident from the available report. | Some concerns: The comparator involved regular physical education exposure, and departures from intended intervention or contamination could not be completely excluded in this behavioral trial context. | Low: Outcome data were not judged sufficiently incomplete to bias the results materially. | Low: Measurement procedures were comparable across groups. | Low: No clear evidence of selective reporting was observed. | Some concerns |
| Zieff et al. (2022) | Low: Randomization appeared acceptable, and baseline characteristics did not suggest serious imbalance. | Low: No important deviations from intended intervention were apparent. | Low: Outcome data were largely complete for the main comparison. | Low: The same outcome assessment approach was used across groups. | Low: No evidence of selective reporting was identified from the available report. | Low |
| Sadeghi et al. (2016) | Low: Randomization appeared acceptable, and baseline BDI-II values were broadly comparable. | Some concerns: Limited detail on adherence monitoring and the non-blinded behavioral nature of the trial left some uncertainty regarding deviations from intended intervention. | Low: No major concern regarding missing outcome data was evident from the available report. | Low: Outcome assessment methods appeared comparable between groups. | Low: No indication of selective reporting was evident. | Some concerns |

# Supplementary 5: Publication bias


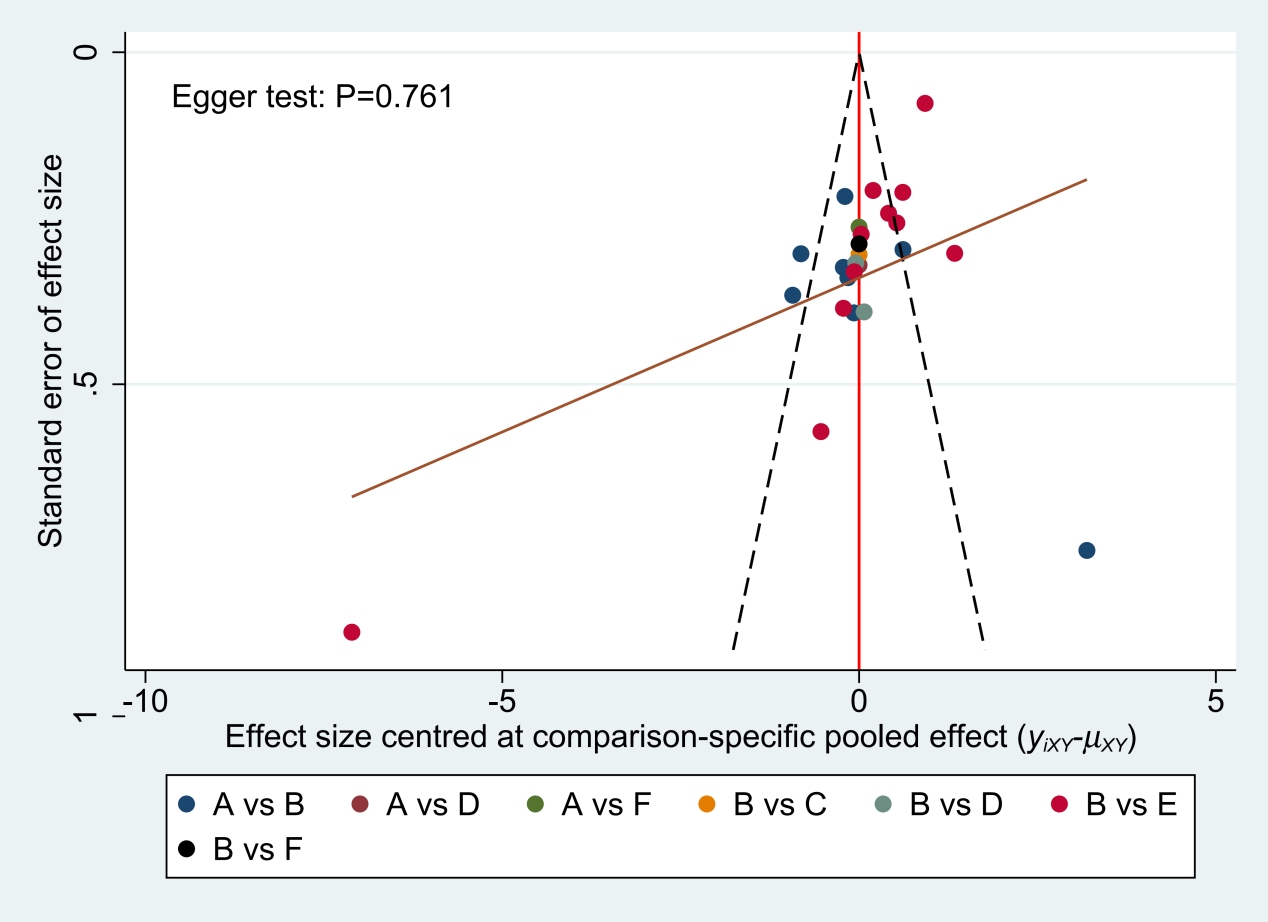


Figure 5.1 The funnel plot of depression. The result of Egger test showed the p=0.761.


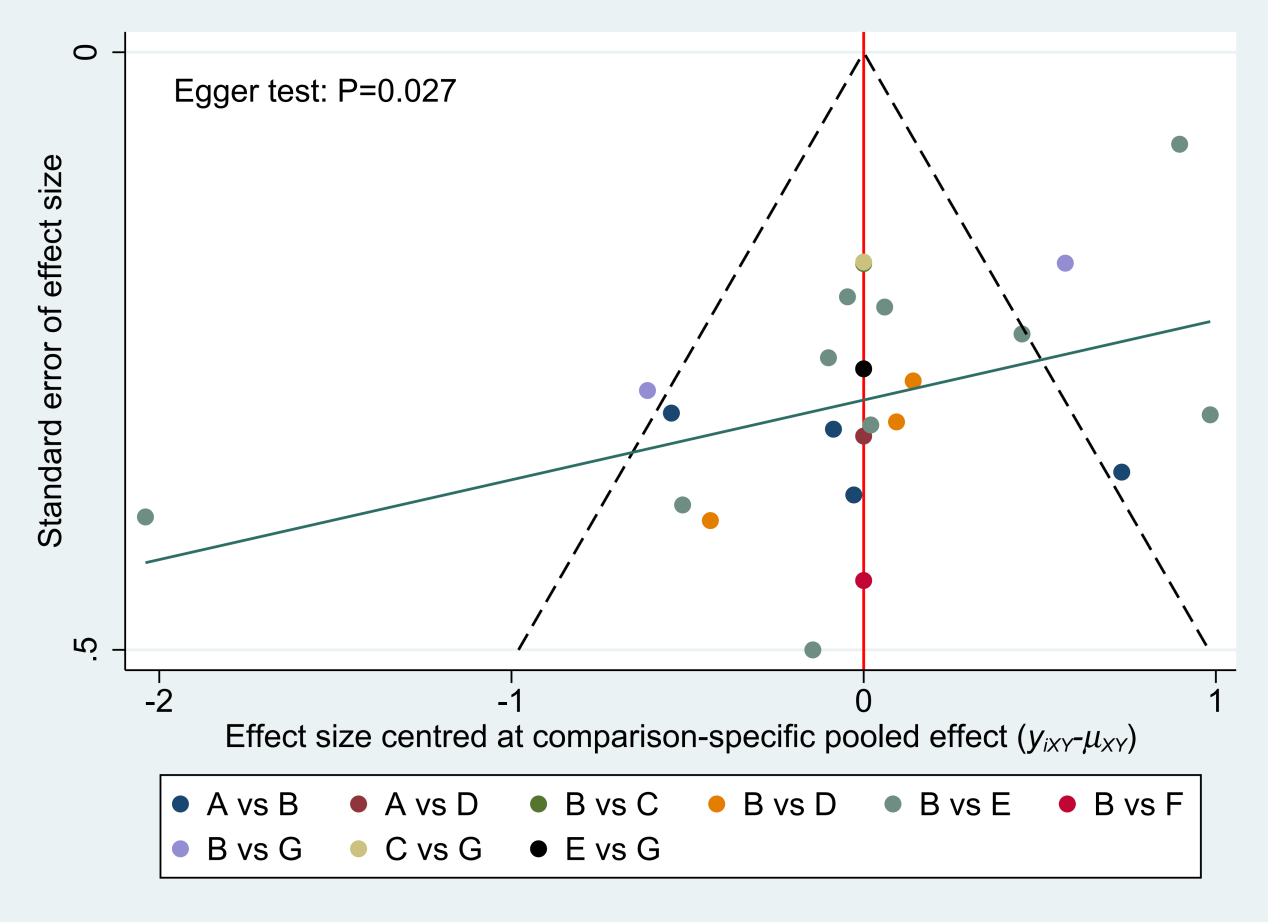


Figure 5.2 The funnel plot of anxiety. The result of Egger test showed the p=0.027.


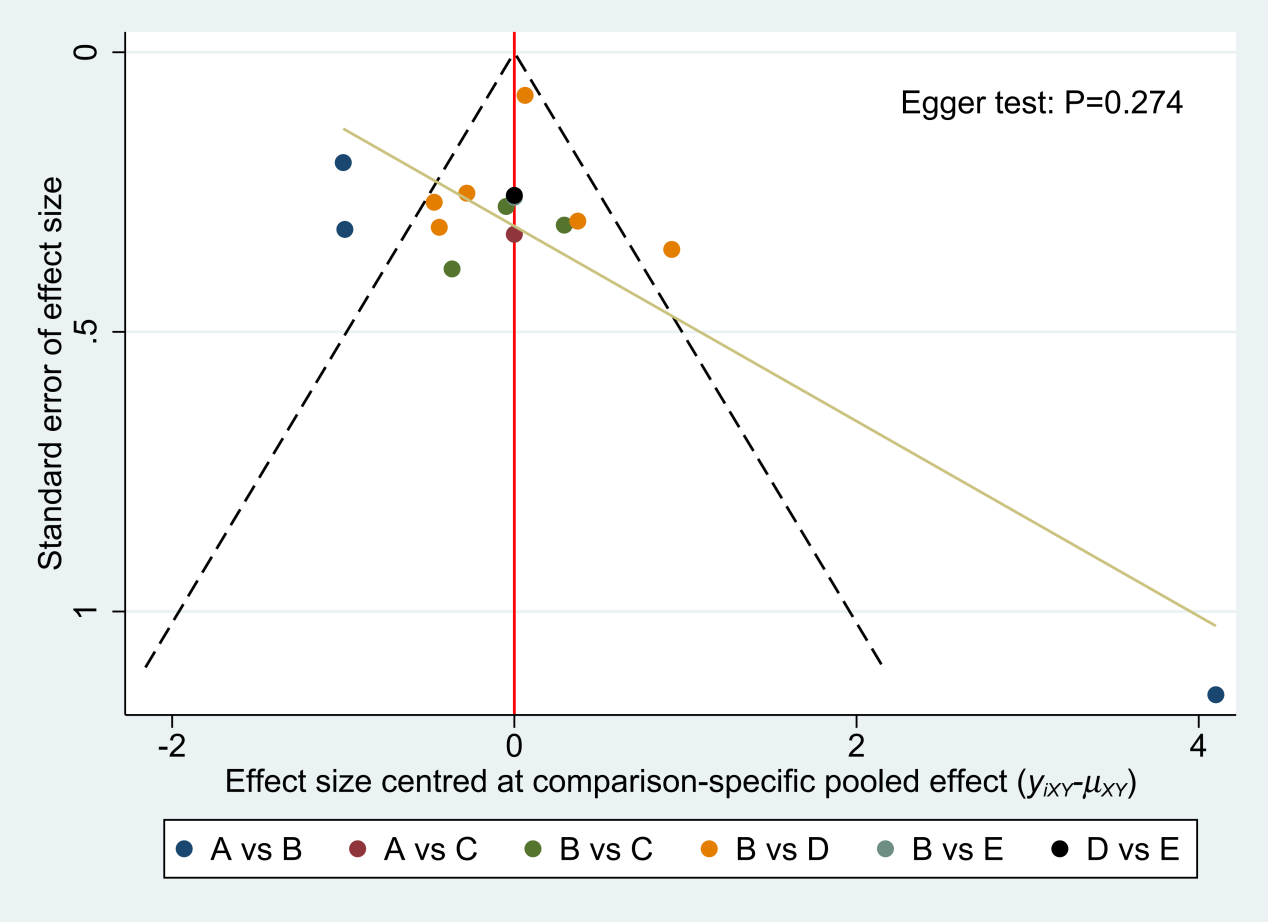


Figure 5.3 The funnel plot of stress. The result of Egger test showed the p=0.274.

# Supplementary 6: Sensitivity analysis


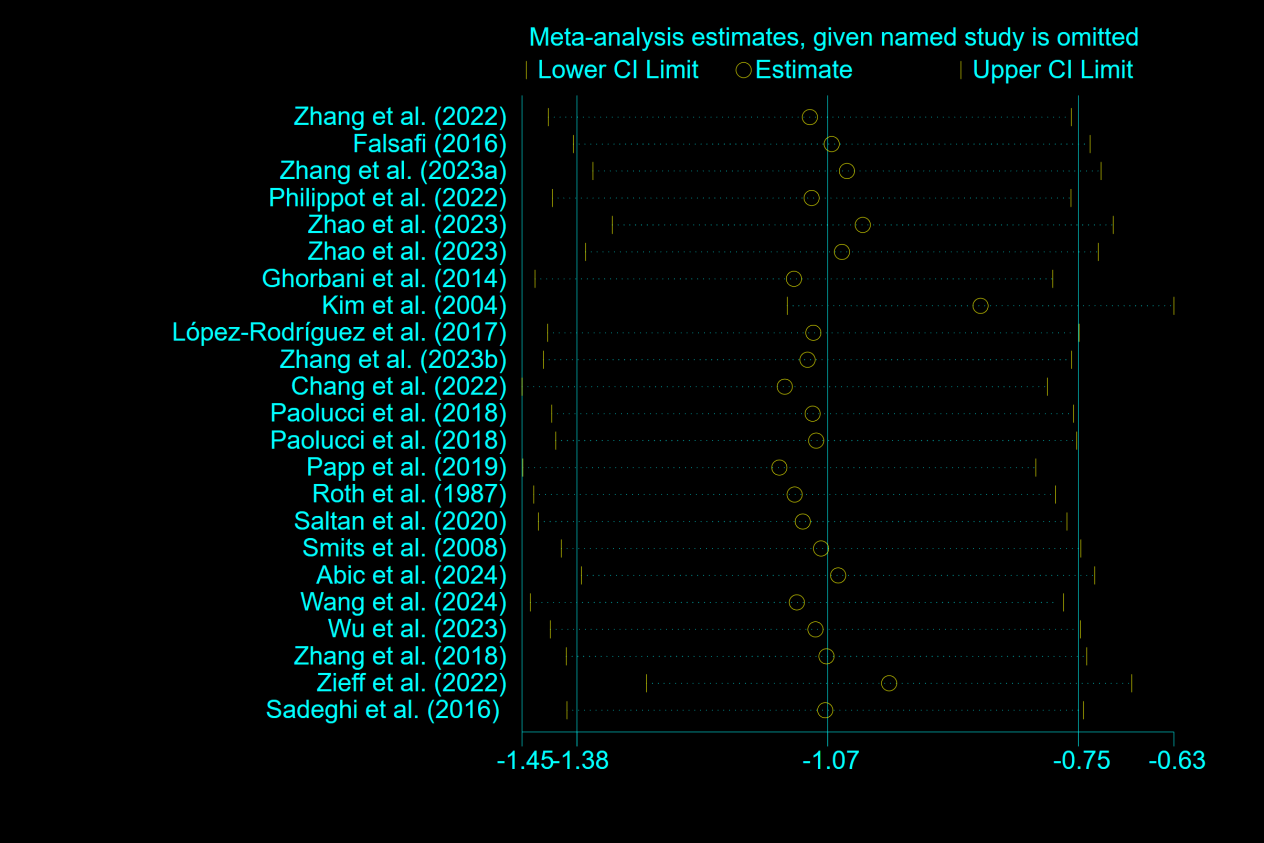


Figure 6.1 Sensitivity analysis plot for depression.


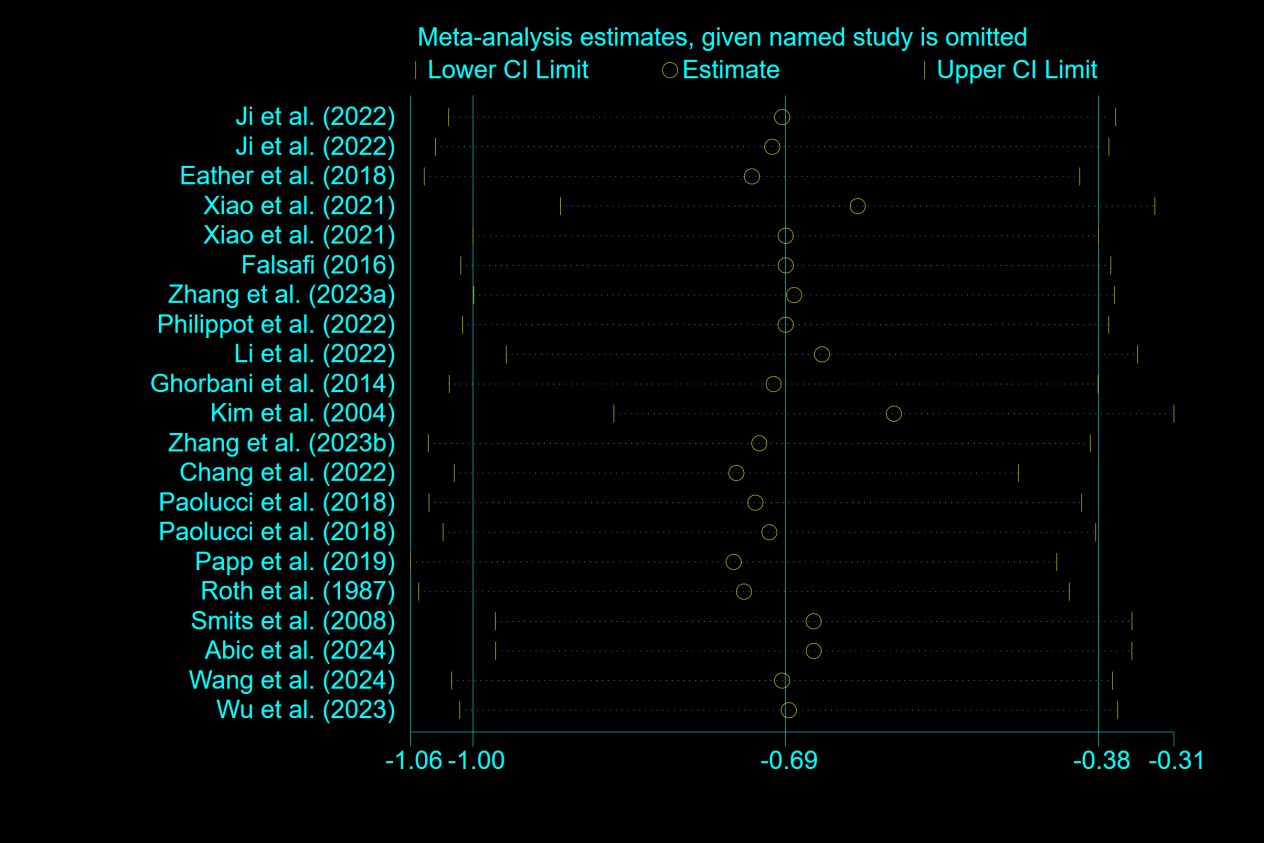


Figure 6.2 Sensitivity analysis plot for anxiety.


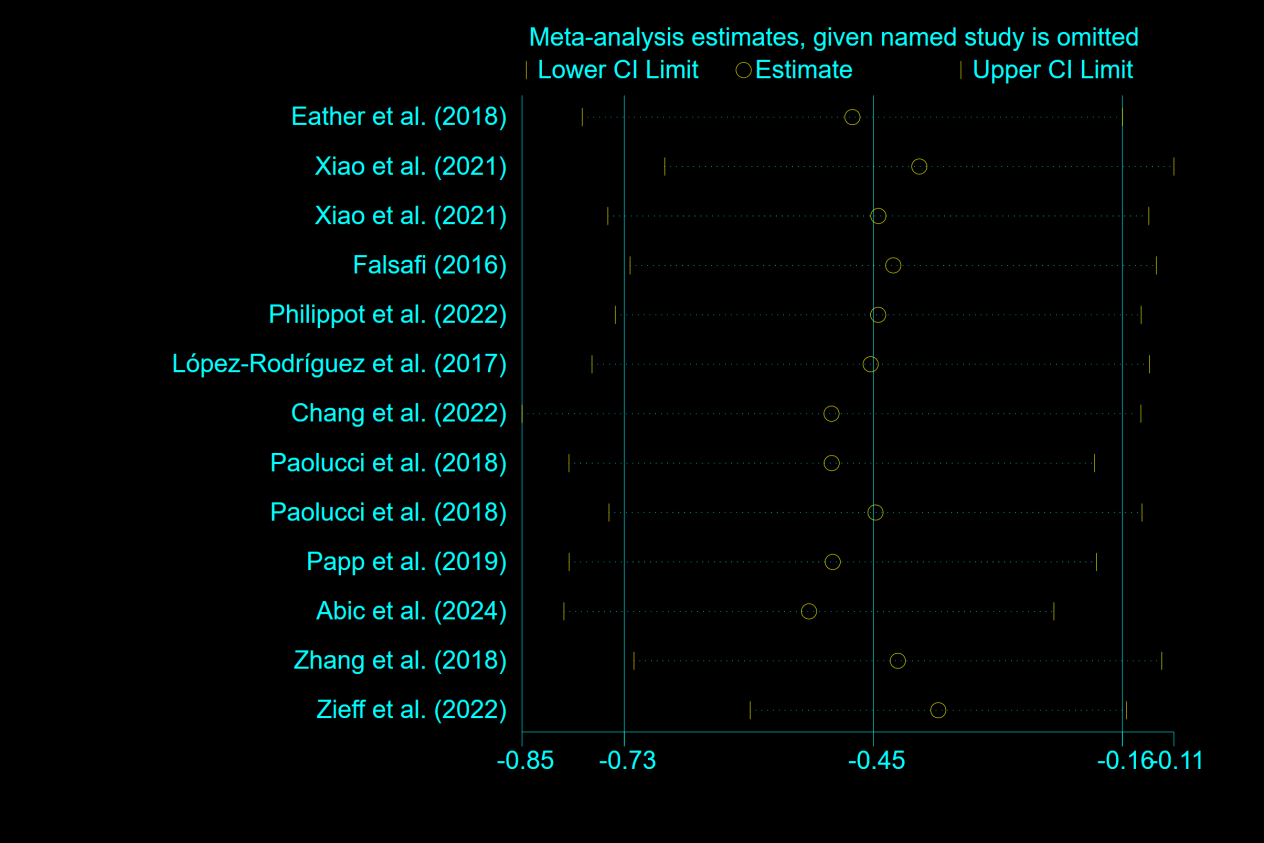


Figure 6.3 Sensitivity analysis plot for stress.

**Table 6.1 Sensitivity analysis excluding the study**

| **Outcome** | **Main analysis in manuscript** | **Sensitivity analysis excluding Zhang et al. (2018)** | **Interpretation** |
| --- | --- | --- | --- |
| Depression | SMD = -1.07, 95% CI -1.38 to -0.75; I² = 87.6% | SMD = -1.08, 95% CI -1.42 to -0.75; I² = 89.0% | Direction and significance unchanged |
| Stress | SMD = -0.45, 95% CI -0.73 to -0.16; I² = 75.2% | SMD = -0.43, 95% CI -0.73 to -0.12; I² = 77.7% | Direction and significance unchanged |

# Supplementary 7: Network meta-regression

| **Outcome** | **Covariate** | **Unit** | **Studies** | **Contrasts** | **Mean covariate** | **Beta** | **95% CI low** | **95% CI high** | **SE** | **P value** |
| --- | --- | --- | --- | --- | --- | --- | --- | --- | --- | --- |
| Depression | Mean age | per 1 year | 21 | 23 | 21.01 | 0.128 | 0.007 | 0.25 | 0.062 | 0.038 |
| Depression | Exercise intensity | per 1 MET | 21 | 23 | 4.783 | 0.083 | -0.148 | 0.314 | 0.118 | 0.479 |
| Depression | Session duration | per 10 min | 21 | 23 | 3.63 | -0.101 | -0.193 | -0.009 | 0.047 | 0.031 |
| Depression | Weekly frequency | per 1 session/week | 21 | 23 | 2.87 | -0.111 | -0.423 | 0.201 | 0.159 | 0.484 |
| Depression | Intervention length | per 1 week | 21 | 23 | 8.13 | -0.046 | -0.094 | 0.003 | 0.025 | 0.067 |
| Depression | Baseline severity ratio | per 0.1 ratio unit | 20 | 22 | 14.578 | -0.043 | -0.092 | 0.006 | 0.025 | 0.083074817 |
| Anxiety | Mean age | per 1 year | 18 | 21 | 21.215 | 0.145 | -0.021 | 0.31 | 0.084 | 0.086592337 |
| Anxiety | Exercise intensity | per 1 MET | 18 | 21 | 5.195 | -0.158 | -0.394 | 0.078 | 0.121 | 0.189727552 |
| Anxiety | Session duration | per 10 min | 18 | 21 | 3.571 | -0.178 | -0.302 | -0.055 | 0.063 | 0.004663111 |
| Anxiety | Weekly frequency | per 1 session/week | 18 | 21 | 2.905 | -0.159 | -0.587 | 0.268 | 0.218 | 0.464710191 |
| Anxiety | Intervention length | per 1 week | 18 | 21 | 7.952 | -0.073 | -0.126 | -0.019 | 0.027 | 0.007371334 |
| Stress | Mean age | per 1 year | 11 | 13 | 21.01 | 0.143 | 0.056 | 0.231 | 0.045 | 0.001340291 |
| Stress | Exercise intensity | per 1 MET | 11 | 13 | 5.046 | -0.097 | -0.201 | 0.007 | 0.053 | 0.068624587 |
| Stress | Session duration | per 10 min | 11 | 13 | 3.885 | -0.049 | -0.103 | 0.005 | 0.028 | 0.074923436 |
| Stress | Weekly frequency | per 1 session/week | 11 | 13 | 2.769 | -0.128 | -0.358 | 0.102 | 0.117 | 0.274550753 |
| Stress | Intervention length | per 1 week | 11 | 13 | 6.923 | -0.031 | -0.091 | 0.029 | 0.031 | 0.309003878 |

# Supplementary 8: Global consistency test


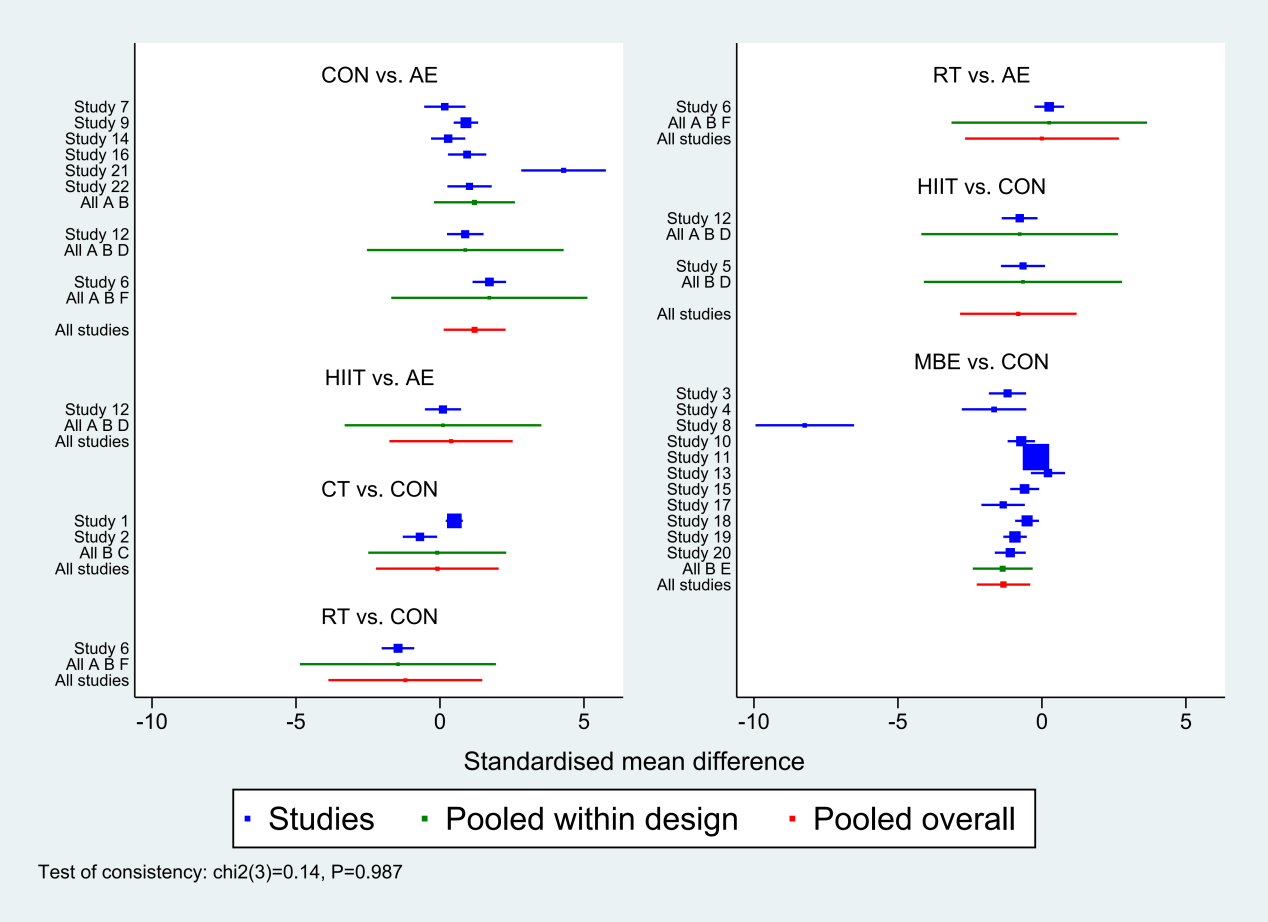


Figure 8.1 The global consistency test of depression. p=0.987.


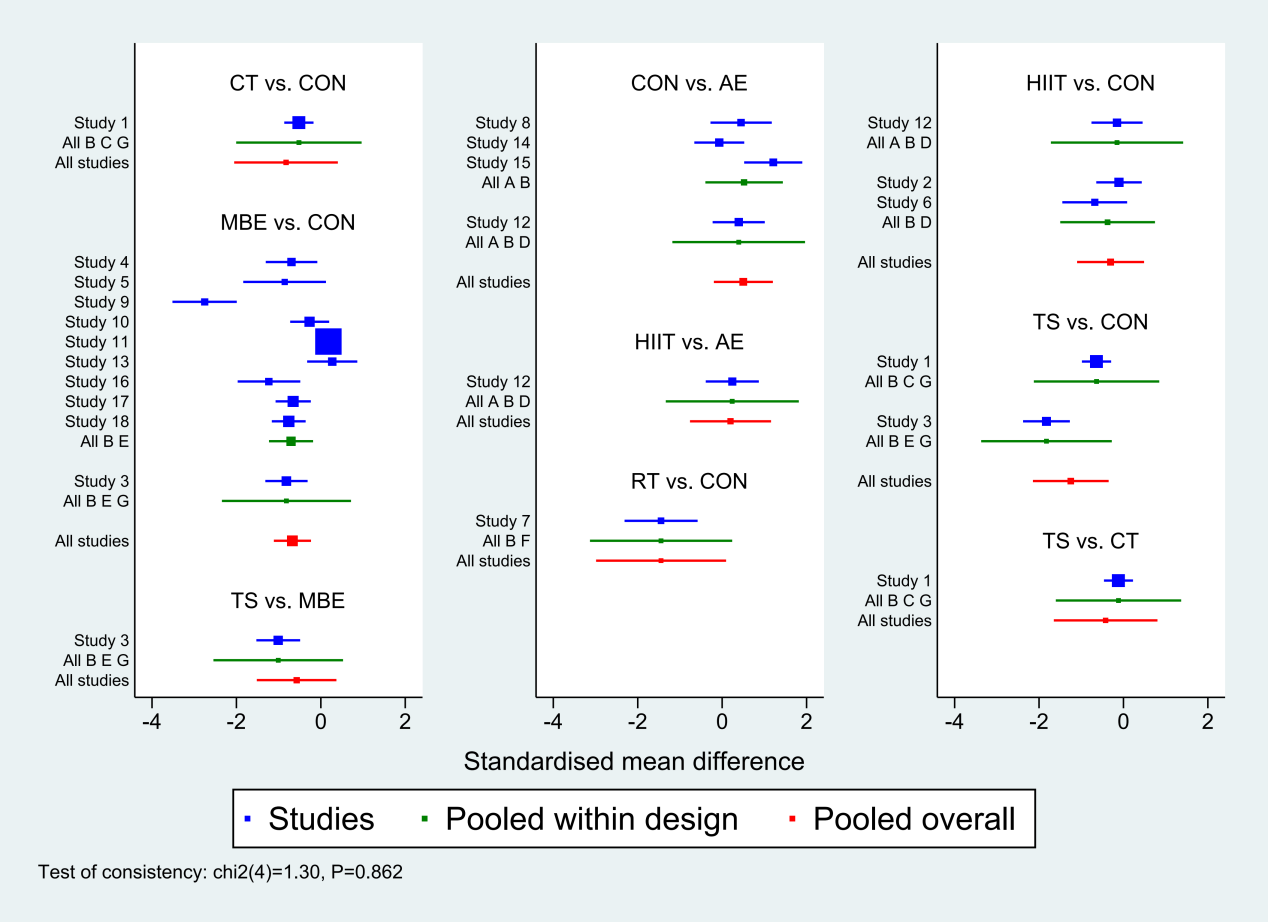


Figure 8.2 The global consistency test of anxiety. p=0.862.


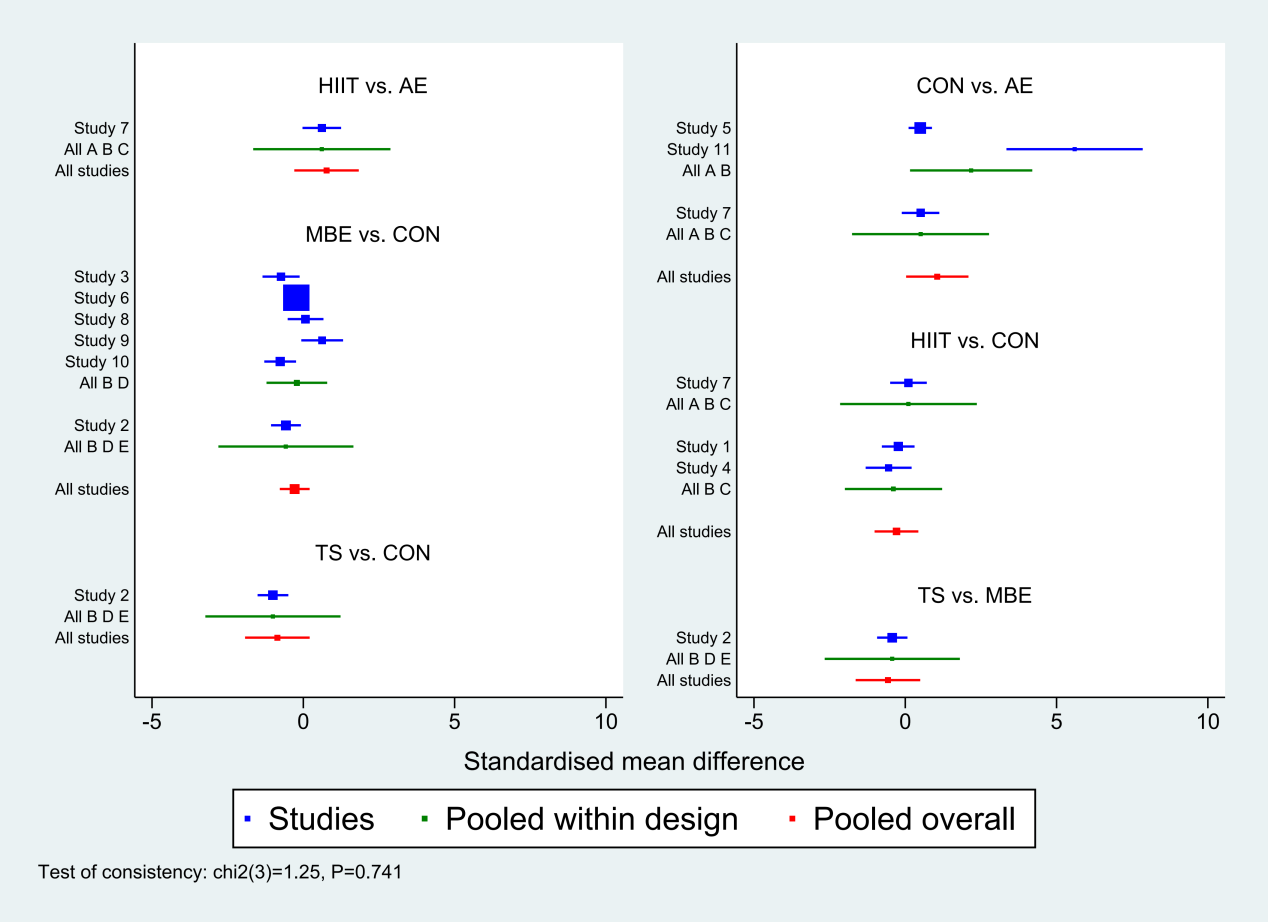


Figure 8.3 The global consistency test of stress. p=0.741.

# Supplementary 9: Results of the nodal splitting

Table 9.1 Results of the nodal splitting for depression

| **Side** | | **Direct** | | **Indirect** | | **Difference** | | **P>\|z\|** | **tau** |
| --- | --- | --- | --- | --- | --- | --- | --- | --- | --- |
|  |  | **Coef.** | **Std. Err.** | **Coef.** | **Std. Err.** | **Coef.** | **Std. Err.** |  |  |
| A | B | 1.222445 | 0.5892722 | 0.6373039 | 3.933487 | 0.5851409 | 3.977326 | 0.883 | 1.618754 |
| A | D | 0.1047854 | 1.661167 | 0.6685994 | 1.644189 | -0.5638139 | 2.337645 | 0.809 | 1.629954 |
| A | F | 0.2553986 | 1.646232 | -0.8989027 | 3.124187 | 1.154301 | 3.532789 | 0.744 | 1.625027 |
| B | C | -0.6950597 | 1.59479 | -2.413984 | 63.31387 | 1.718924 | 63.33395 | 0.978 | 1.565421 |
| B | D | -0.7164991 | 1.179731 | -1.558328 | 3.142153 | 0.8418286 | 3.356655 | 0.802 | 1.630013 |
| B | E | -1.340526 | 0.4879115 | -2.455822 | 19.12045 | 1.115296 | 19.12655 | 0.954 | 1.565615 |
| B | F | -1.459421 | 1.65046 | -0.3051193 | 3.117488 | -1.154301 | 3.532789 | 0.744 | 1.625027 |

Table 9.3 Results of the nodal splitting for anxiety

| Side | | Direct | | Indirect | | Difference | | P>\|z\| | tau |
| --- | --- | --- | --- | --- | --- | --- | --- | --- | --- |
|  |  | Coef. | Std. Err. | Coef. | Std. Err. | Coef. | Std. Err. |  |  |
| A | B | 0.4872086 | 0.3774876 | 0.8186261 | 1.645317 | -0.3314175 | 1.686958 | 0.844 | 0.6765571 |
| A | D | 0.2409769 | 0.7504238 | 0.1618321 | 0.6880164 | 0.0791448 | 1.018251 | 0.938 | 0.6782311 |
| B | C | -0.5200701 | 0.6592745 | -2.780494 | 1.672722 | 2.260424 | 1.798136 | 0.209 | 0.6351263 |
| B | D | -0.297803 | 0.4345795 | -0.4083577 | 1.56703 | 0.1105547 | 1.625376 | 0.946 | 0.6782997 |
| B | E | -0.7060062 | 0.222215 | 1.53615 | 1.77669 | -2.242156 | 1.791029 | 0.211 | 0.634889 |
| B | F | -1.446641 | 0.7861676 | -1.059358 | 63.25938 | -0.3872833 | 63.26416 | 0.995 | 0.6501534 |
| B | G | -1.20276 | 0.5081426 | -1.587702 | 1.349685 | 0.3849414 | 1.44024 | 0.789 | 0.6794749 |
| C | G | -0.1176092 | 0.6589489 | -2.379177 | 1.673516 | 2.261568 | 1.798509 | 0.209 | 0.6351386 |
| E | G | -0.9999875 | 0.7134735 | -0.1998859 | 0.668708 | -0.8001017 | 0.9749208 | 0.412 | 0.6625516 |

Table 9.3 Results of the nodal splitting for stress

| Side | | Direct | | Indirect | | Difference | | P>\|z\| | tau |
| --- | --- | --- | --- | --- | --- | --- | --- | --- | --- |
|  |  | Coef. | Std. Err. | Coef. | Std. Err. | Coef. | Std. Err. |  |  |
| A | B | 1.206168 | 0.6907776 | 1.462538 | 1.755228 | -0.2563697 | 1.888792 | 0.892 | 0.7252652 |
| A | C | 0.6139799 | 0.8711342 | 1.354759 | 1.124091 | -0.7407794 | 1.422646 | 0.603 | 0.8080393 |
| B | C | -0.2182557 | 0.5007701 | -2.504407 | 2.356297 | 2.286151 | 2.405423 | 0.342 | 0.8038985 |
| B | D | -0.2838218 | 0.2520202 | -2.051923 | 25.85216 | 1.768102 | 25.85374 | 0.945 | 0.555006 |
| B | E | -1.002558 | 0.7699247 | -0.2935254 | 1.499313 | -0.7090324 | 1.684233 | 0.674 | 0.7249455 |
| D | E | -0.4288178 | 0.768913 | -1.139165 | 1.501345 | 0.7103474 | 1.68457 | 0.673 | 0.7250465 |

# Supplementary 10: GRADE certainty for the network comparisons

| **Outcome** | **Main network comparison** | **Network estimate**  **(SMD, 95% CI)** | **Risk of bias** | **Inconsistency** | **Indirectness** | **Imprecision** | **Publication bias** | **Overall certainty** |
| --- | --- | --- | --- | --- | --- | --- | --- | --- |
| Depression | MBE vs CON | -1.34 (-2.30 to -0.39) | Not serious | Serious | Not serious | Serious | Undetected | Low |
| Depression | AE vs CON | -1.21 (-2.31 to -0.10) | Not serious | Serious | Not serious | Serious | Undetected | Low |
| Anxiety | MBE vs CON | -0.67 (-1.11 to -0.23) | Not serious | Serious | Not serious | Serious | Serious | Very low |
| Anxiety | TS vs CON | -1.25 (-2.14 to -0.35) | Not serious | Serious | Not serious | Very serious | Serious | Very low |
| Stress | AE vs CON | -1.06 (-2.09 to -0.03) | Not serious | Serious | Not serious | Serious | Undetected | Low |
